# Comprehensive association analysis of speech recognition thresholds after cisplatin‐based chemotherapy in survivors of adult‐onset cancer

**DOI:** 10.1002/cam4.5218

**Published:** 2022-09-12

**Authors:** Mohammad Shahbazi, Xindi Zhang, Paul C. Dinh, Victoria A. Sanchez, Matthew R. Trendowski, Megan M. Shuey, Tessa Nguyen, Regeneron Genetics Center, Darren R. Feldman, David J. Vaughn, Chunkit Fung, Christian Kollmannsberger, Neil E. Martin, Lawrence H. Einhorn, Nancy J. Cox, Robert D. Frisina, Lois B. Travis, Mary Eileen Dolan

**Affiliations:** ^1^ Department of Medicine University of Chicago Chicago Illinois USA; ^2^ Department of Medical Oncology Indiana University Indianapolis Indiana USA; ^3^ Department of Otolaryngology—Head and Neck Surgery University of South Florida Tampa Florida USA; ^4^ Department of Medicine and Vanderbilt Genetics Institute, Vanderbilt University Medical Center Nashville Tennessee USA; ^5^ Center for Audiology, Speech, Language and Learning Northwesthern University Chicago Illinois USA; ^6^ Department of Medical Oncology, Memorial Sloan‐Kettering Cancer Center New York New York USA; ^7^ Department of Medicine University of Pennsylvania Philadelphia Pennsylvania USA; ^8^ J.P. Wilmot Cancer Institute, University of Rochester Medical Center Rochester New York USA; ^9^ Division of Medical Oncology University of British Columbia Vancouver British Columbia Canada; ^10^ Department of Radiation Oncology Dana‐Farber Cancer Institute Boston Massachusetts USA; ^11^ Departments of Medical Engineering and Communication Sciences and Disorders, Global Center for Hearing and Speech Research University of South Florida Tampa Florida USA

**Keywords:** chemotherapy, GWAS, ototoxicity, speech recognition

## Abstract

**Purpose:**

Deficits in speech understanding constitute one of the most severe consequences of hearing loss. Here we investigate the clinical and genetic risk factors for symmetric deterioration of speech recognition thresholds (SRT) among cancer survivors treated with cisplatin.

**Methods:**

SRT was measured using spondaic words and calculating the mean of measurements for both ears with symmetric SRT values. For clinical associations, SRT‐based hearing disability (SHD) was defined as SRT≥15 dB hearing loss and clinical variables were derived from the study dataset. Genotyped blood samples were used for GWAS with rank‐based inverse normal transformed SRT values as the response variable. Age was used as a covariate in association analyses.

**Results:**

SHD was inversely associated with self‐reported health (*p =* 0.004). Current smoking (*p* = 0.002), years of smoking (*p* = 0.02), BMI (*p* < 0.001), and peripheral motor neuropathy (*p =* 0.003) were positively associated with SHD, while physical activity was inversely associated with SHD (*p* = 0.005). In contrast, cumulative cisplatin dose, peripheral sensory neuropathy, hypertension, and hypercholesterolemia were not associated with SHD. Although no genetic variants had an association *p* value < 5 × 10^−8^, 22 genetic variants were suggestively associated (*p* < 10^−5^) with SRT deterioration. Three of the top variants in 10 respective linkage disequilibrium regions were either positioned within the coding sequence or were eQTLs for genes involved in neuronal development (*ATE1*, *ENAH,* and *ZFHX3*).

**Conclusion:**

Current results improve our understanding of risk factors for SRT deterioration in cancer survivors. Higher BMI, lower physical activity, and smoking are associated with SHD. Larger samples would allow for expansion of the current findings on the genetic architecture of SRT.

## Graphical abstract

We investigated the risk factors associated with decline in understanding speech as measured by speech recognition threshold among testicular cancer survivors. Associated factors were higher BMI, lower physical activity and smoking, and peripheral motor neuropathy alongside genetic variants with suggestive associations including those related to three genes involved in neuronal development.

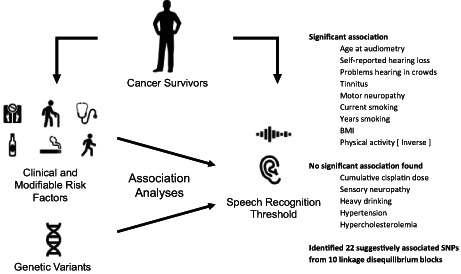

## 1 INTRODUCTION

The number of cancer survivors is estimated at 18 million by 2022,^1^ providing impetus to investigate the emergence of disabilities and their risk factors in this growing population. Cisplatin, a first‐line treatment for a wide range of cancers, is associated with hearing loss,^2^, ^3^ tinnitus,^4^ peripheral neuropathy,^5^ nephrotoxicity,^6^ gastrointestinal toxicity,^7^ and myelosuppression.^8^, ^9^

Hearing loss is a disability with direct impact on quality of life.^10^, ^11^ One of the most debilitating consequences of hearing loss is deterioration in understanding speech which can be quantified by measuring a speech recognition threshold (SRT). The SRT is measured by determining the softest level at which an individual can hear and accurately repeat back familiar two‐syllable spondaic words. The SRT is usually closely correlated with pure‐tone average (PTA) at low frequencies (0.5–2 kHz).^12^, ^13^

A primary cause of cisplatin‐induced hearing loss is believed to be irreversible damage to the outer hair cells of the cochlea.^14^ The ototoxic effects of cisplatin chemotherapy as measured by PTA have demonstrated that hearing loss is maximal in the high frequency range.^2^, ^15^, ^16^, ^17^, ^18^ Depending on the mode of evaluation, the reported incidence of hearing loss following cisplatin therapy varies considerably. For example, the frequency of self‐reported hearing loss using a questionnaire is significantly lower compared with PTA which takes into account measurements of hearing at high frequencies.^2^, ^19^, ^20^ The clinical relevance of cisplatin‐induced high frequency hearing loss in survivors of adult‐onset cancer has been debated. In our previous study,^21^ we demonstrated that deficits restricted to high frequency hearing loss (10–12 kHz) are perceptible in about 1 in 4 of all survivors after cisplatin‐based chemotherapy.

In the current investigation, data from The Platinum Study,^22^ a comprehensive multi‐institutional collaboration, were used in a cross sectional study to examine the association of age and cisplatin dose with SRT deterioration in cisplatin‐treated cancer survivors. Further, we evaluated associations of clinical and genetic factors with SRT deficits.

## 2 MATERIALS AND METHODS

### 2.1 Study subjects

Testicular cancer survivors with serologically or histologically confirmed diagnosis of germ cell tumor visiting the clinic for follow‐up were enrolled in the Platinum Study. Chemotherapy consisted of cisplatin‐based regimens; survivors did not have prior chemotherapy for other cancers and completed chemotherapy more than a year prior to the enrollment. Enrolled survivors completed comprehensive health questionnaires, underwent brief physical examination and were audiometrically evaluated. The Platinum Study involves eight medical centers in the United States, Canada, and United Kingdom.^23^

### 2.2 SRT measurements

Hearing was evaluated by a licensed audiologist conducted in a clinical setting, inside a sound‐treated booth using calibrated equipment. Pure‐tone and speech audiometry was obtained for each ear following professional standards.^24^ The speech recognition threshold (SRT) was measured using standardized recorded spondaic words (CID W‐1).^25^ Spondaic words are two‐syllable words, with equal vocal stress on each syllable, and considered to be equally familiar in American English (e.g., baseball, cowboy, hotdog).^25^ Subjects were instructed to repeat back the words heard. The audiologist determined if the word recognized and repeated by the subject was correct or incorrect before presenting the next word at an adjusted presentation level to converge on the threshold using the standard clinical method known as the modified Hughson–Westlake procedure.^26^

Figure S1 outlines quality control steps for SRT measurements. From 2740 SRT measurements, 27 had duplicated mismatching entries. These duplicated entries were resolved by calculating the respective PTAs at 0.5, 1, and 2 kHz and keeping the SRT measurement that was more closely aligned with the PTA. Both mismatching SRT measurements were removed if they had more than 5 dB hearing level distance from the PTA or were equally distanced from it.

**FIGURE 1:**
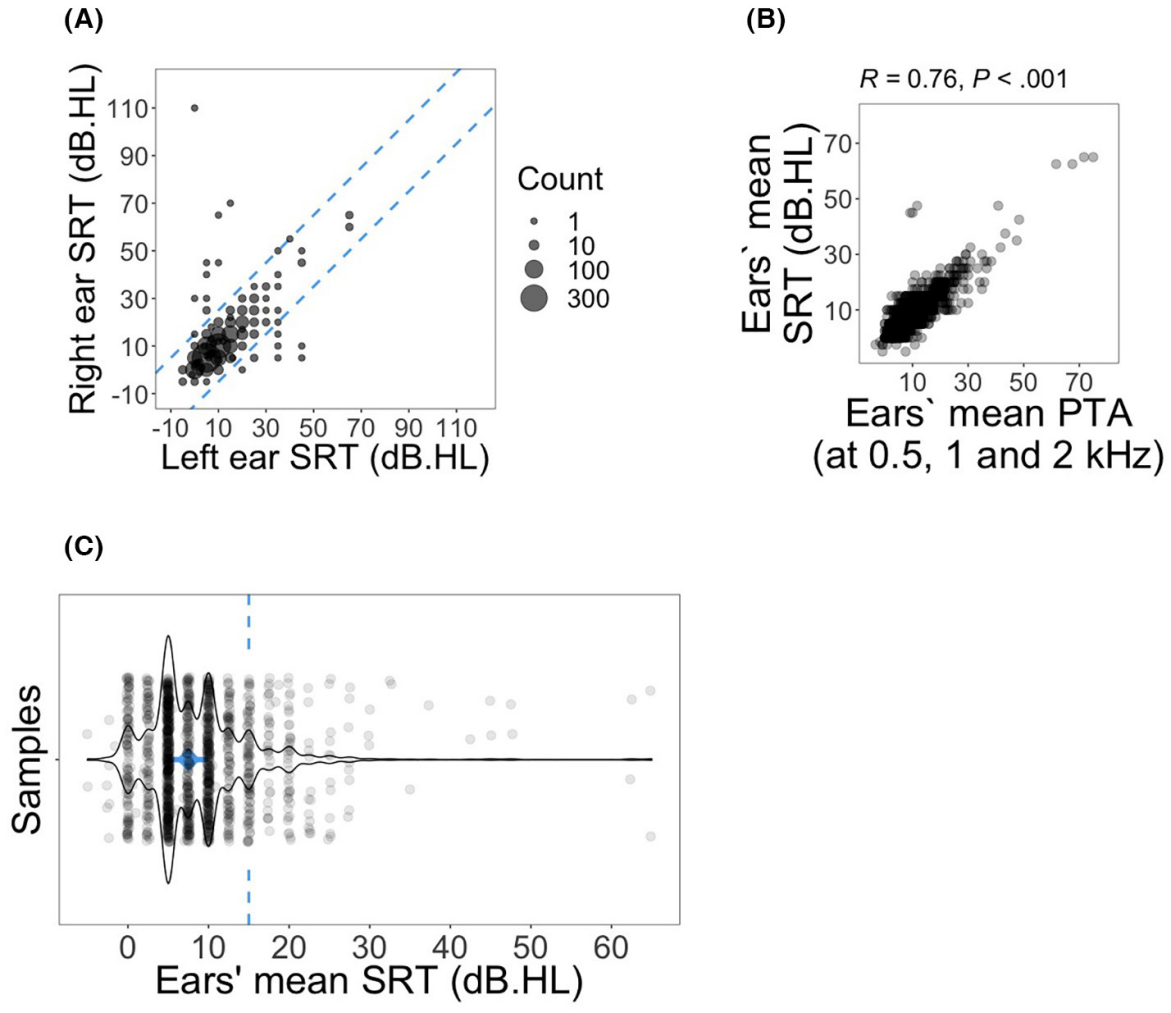
Criteria to define SRT‐based hearing disability groups. (A) Comparison of SRT values between ears. Subjects with more than 15 dB hearing level difference in SRT measurements between ears were excluded from further analysis. Dotted line, 15 dB hearing level margins. (B) Comparison of ears' mean SRT with ears' mean PTA at 0.5, 1, and 2 kHz across samples (*n* = 1343). Spearman's rank correlation coefficient and *p* value are provided. (C) Distribution of ears' mean SRT measurements (*n* = 1347). Dashed line, 15 dB hearing level cutoff for hearing disability; vertical line, interquartile region; square point, median

For each subject, SRT values for left and right ears were compared (Figure 1A) and subjects with measurements for only a single ear or with asymmetric SRT measurements (more than 15 dB hearing level difference between ears) were excluded from all further analyses. Next, for each subject, the arithmetic mean of SRT measurements for both ears was calculated.

### 2.3 Clinical variables and association analysis

To obtain the dose of administered cisplatin, chemotherapy treatment regimens and/or individual chemotherapy cycles were abstracted from the subjects' medical records. Cumulative cisplatin dosages were calculated for standard treatment regimens or from individual chemotherapy cycles for nonstandard treatment regimens. If necessary and depending on data availability, dosages were converted to mg/m^2^ with body surface area (m^2^). When not available, body surface area or chemotherapy cycle details were imputed to meet the expected standard cisplatin cycle dosage (100 mg/m^2^ per cycle).

For clinical associations besides age and dose of cisplatin, subjects were excluded from the analysis if evaluations and audiometry were done more than 1.2 years (438 days) apart. Age was calculated as the difference in days between date of birth and date of data collection, divided by 365 and rounded to the nearest integer. The association of age with hearing loss is well documented.^27^, ^28^ To control for the effect of age on clinical associations, age was included as a covariate (except for the association with age itself).

Distribution and preparation of clinical variables used for clinical association analyses of “self‐reported hearing loss,” “problem hearing in crowds,” tinnitus, peripheral motor neuropathy, peripheral sensory neuropathy, heavy drinking, ever smoking, current smoking, hypertension and hypercholesterolemia are outlined in Figures S2–S5. Other than BMI, audiometry data and cumulative cisplatin dosages, all clinical variables used for the association analyses were self‐reported. European organization for research and treatment of cancer (EORTC) questionnaire for chemotherapy‐induced peripheral neuropathy (CIPN20) questions were used for evaluation of peripheral neuropathies (Table S1). EORTC‐CIPN20 has been validated.^29^ Furthermore, both questions regarding self‐reported hearing loss were obtained from EORTC‐CIPN20 and validated scale for chemotherapy‐induced long‐term neuropathy (SCIN) questionnaires.^30^ One of the two questions regarding tinnitus was also obtained from SCIN. Physical activities were calculated as metabolic equivalent of task (MET) using questionnaire responses as described earlier.^31^

**FIGURE 2:**
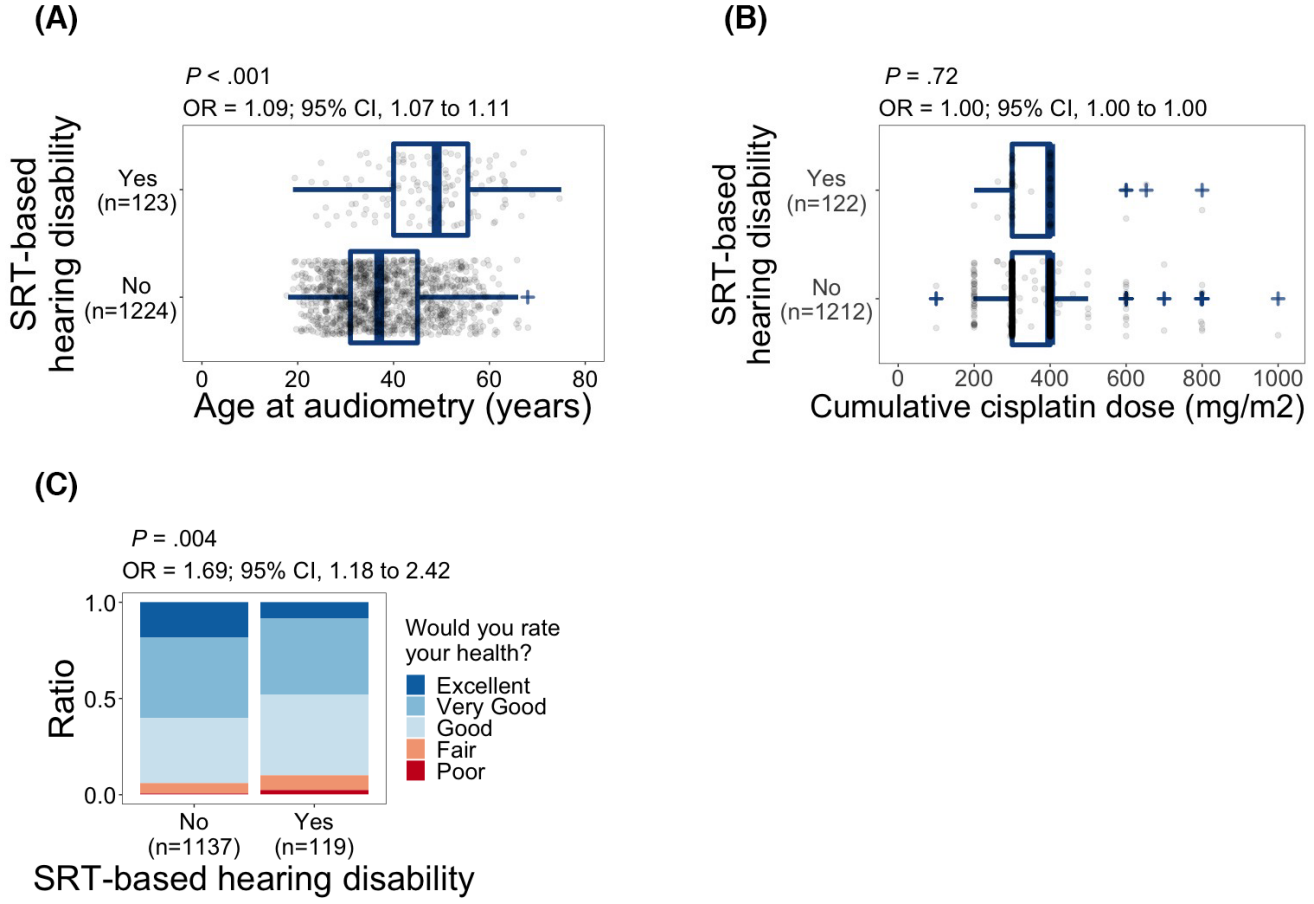
Distribution of SRT‐based hearing disability relative to (A) age at audiometry, (B) cumulative cisplatin dose, and (C) self‐reported health. The question and respective answers regarding self‐reported health are shown in (C). *p* values and odds ratios (OR) are calculated using logistic regression model (A, B) or ordinal logistic regression (C). Model for (B): hearing disability = cumulative cisplatin dose + age at audiometry. Model for (C): self‐reported health = hearing disability + age at health evaluation

**FIGURE 3:**
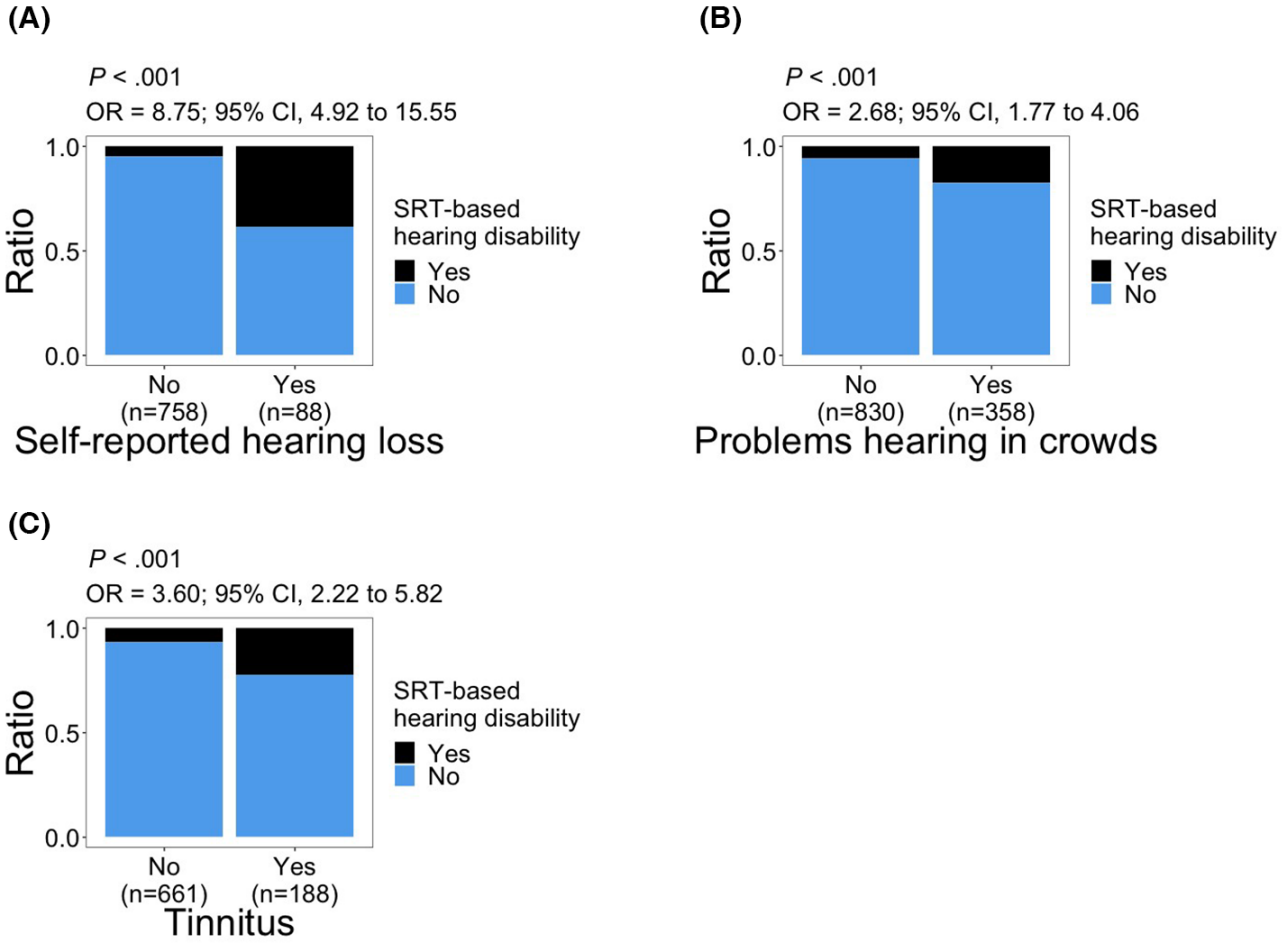
Distribution of SRT‐based hearing disability relative to (A) self‐reported hearing loss, (B) problems hearing in crowds, and (C) tinnitus. *p* values and odds ratios (OR) are calculated using logistic regression model: hearing disability = response + age at audiometry

**FIGURE 4:**
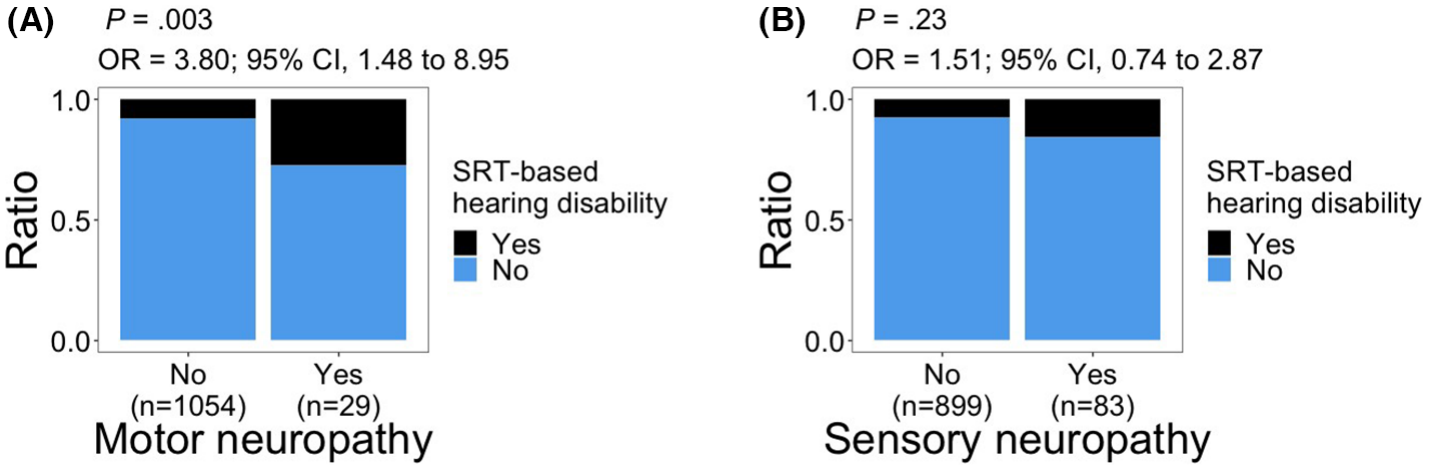
Distribution of SRT‐based hearing disability relative to (A) peripheral motor neuropathy and (B) peripheral sensory neuropathy. *p* values and odds ratios (OR) are calculated using logistic regression model: hearing disability = response + age at audiometry

**FIGURE 5:**
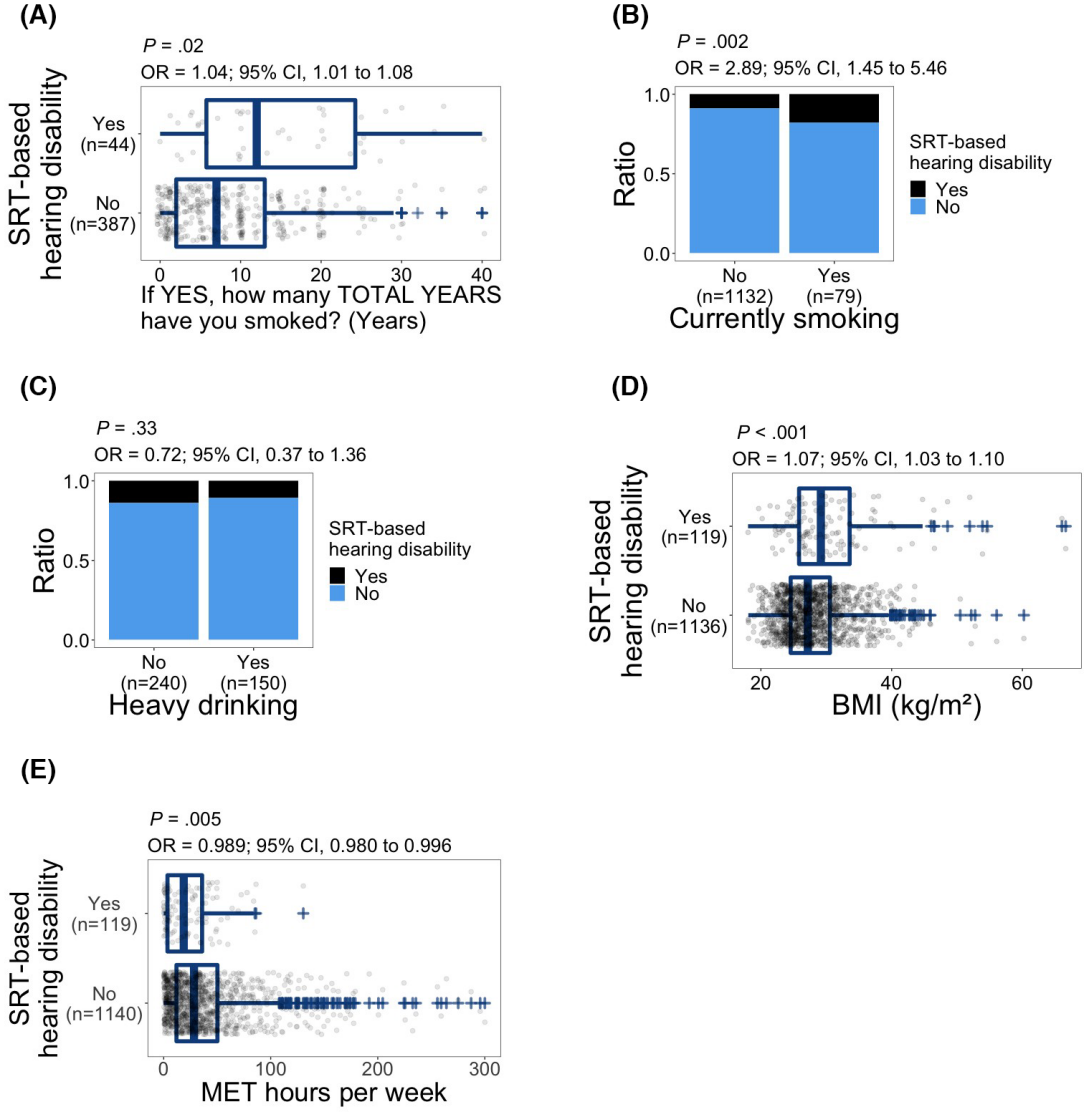
Distribution of SRT‐based hearing disability relative to (A) years of smoking, (B) currently smoking, (C) heavy drinking, (D) BMI, and (E) physical activity. *p* values and odds ratios (OR) are calculated using logistic regression model: hearing disability = response + age at audiometry. In (A) question regarding years of smoking followed the question ‘Have you EVER smoked cigarettes?’, presented in Figure S4B

For clinical association analyses, calculated SRTs were transformed to binary with values equal to or greater than 15 dB hearing level placed in the hearing disability group. Ordinal logistic regression from MASS package^32^ (https://CRAN.R‐project.org/package=MASS; RRID:SCR_019125) was used for association of SRT‐based hearing disability with self‐reported health. Logistic regression was used for other clinical association analyses with SRT‐based hearing disability. For the number of subjects in clinical associations refer to Figure S1 or the respective association figures (Figures 2, 3, 4, 5).

### 2.4 Preparation of genotype data

During the clinical evaluation, peripheral blood samples were collected from subjects and were used for DNA extraction. Genotyping was performed by Regeneron Pharmaceuticals using Infinium Global Screening Array v1.0 (Illumina Inc.).

Genotype data with matching SRT measurements were subject to quality control steps outlined in Figures S6–S9. Briefly, duplicate variants were excluded and variants and samples were filtered for missing call rates using a relaxed (>0.2) and a stringent (>0.02) threshold subsequently. X chromosome homozygosity higher than 0.8 was used to exclude subjects with sex discrepancy (Figure S6). Next, the autosomal variants were selected and genotypes were anchored by 1000 Genomes data^33^ (available at: ftp://ftp‐trace.ncbi.nih.gov/1000genomes/ftp/release/20100804/ALL.2of4intersection.20100804.genotypes.vcf.gz) to select for subjects with European ancestry. Variants with minor allele frequency (MAF) lower than 0.05 were filtered out (Figure S7). Deviating variants from Hardy–Weinberg equilibrium (HWE; *p* < 10^−6^) were excluded. Subjects that deviated from the mean heterozygosity rate by more than three standard deviations were excluded. For filtration based on related genotypes, pi‐hat threshold of 0.0625 was used to select related subjects. Related pairs were determined based on mean identity‐by‐descent (IBD; pi‐hat >0.0625) and in each related pair, the subject with higher missing call rate was excluded (Figure S8). The resulting genotypes from previous steps were used for imputation through Michigan imputation server^34^ (https://imputationserver.sph.umich.edu/, RRID:SCR_017579). bgzip from HTSlib^35^ (https://www.htslib.org, RRID:SCR_002105) and 7‐Zip (https://www.7‐zip.org, SCR_021270) were used for compression and decompression of genotype data prior and post imputation. Imputed genotypes were filtered to keep variants with an imputation *R* ^2^ above 0.8 and to remove variants with MAF below 0.05 or with deviation from HWE (*p* < 10^−6^) (Figure S9A).

### 2.5 Genome‐wide association analysis

To prepare the SRT data for subjects with matching quality‐controlled genotype, the arithmetic mean of SRT measurements for both ears was subjected to rank‐based inverse normal transformation (Figure S9B) using GenABEL package^36^ (https://CRAN.R‐project.org/package=GenABEL, RRID:SCR_001842). The transformed SRT data were used for linear regression‐based genome‐wide association analysis. Inclusion of the 10 genetic principal components is a common practice to control for population stratification.^3^, ^37^, ^38^, ^39^ Consistently, the top 10 principal components and the biological age during audiometry used as covariates. PLINK,^40^ V1.90b6.21 (www.cog‐genomics.org/plink/1.9/, RRID:SCR_021271) which includes implementations of earlier method^41^, ^42^, ^43^ was used in quality control steps, principal component analysis and genome‐wide association analysis. Regression was repeated for selected target SNPs using R to obtain support statistical information. The NCBI database of genetic variation^44^ (https://www.ncbi.nlm.nih.gov/snp/) was used to check the details of target variants. LocusZoom webtool,^45^ V0.13.2 (https://my.locuszoom.org, RRID:SCR_021374) was used to generate regional Manhattan plots with LD population set to European. GTEx portal (https://gtexportal.org/home/, RRID:SCR_001618) was used to check if identified SNPs are known expression quantitative trait loci (eQTLs) or splicing quantitative trait loci (sQTLs).

### 2.6 Software information

Bash (https://www.gnu.org/software/bash/, SCR_021268), R (http://www.r‐project.org/, RRID:SCR_001905), and RStudio (http://www.rstudio.com/, RRID:SCR_000432) provided the analysis environment. Packages dplyr (https://CRAN.R‐project.org/package=dplyr, RRID:SCR_016708), tidyr^46^ (https://CRAN.R‐project.org/package=tidyr, RRID:SCR_017102), purrr (https://CRAN.R‐project.org/package=purrr, SCR_021267), sas7bdat (https://CRAN.R‐project.org/package=sas7bdat) were used for data handling in the analysis pipeline. ggplot2^47^ (https://ggplot2.tidyverse.org, RRID:SCR_014601) was used for plot generation. Rcartocolor (https://nowosad.github.io/rcartocolor, SCR_021269) provided the color palette used for genetic ancestry plot.

## 3 RESULTS

For this cohort, demographic information is provided in Table 1. The median age at diagnosis was 31 years old and age at audiometry was 37 years old. The majority of subjects were white (82.3%) and 68.2% were college‐educated. To check for the consistency of the data, both ears' mean SRTs were plotted against arithmetic mean of ears' PTAs at 0.5, 1 and 2 kHz (Figure 1A). Spearman's rho test confirmed a strong and significant correlation between SRT and PTA at 0.5, 1, and 2 kHz (*R* = 0.76; *p* < 0.001; Figure 1B). Four subjects without PTA measurements were excluded from the plot. SRTs were transformed to binary with values equal to or greater than 15 dB hearing level placed in the hearing disability group and those less than 15 dB as controls (Figure 1C).

**TABLE 1:** Demographic features of subjects with symmetric SRT measurements

Characteristic	*n* (%)
Age at audiometry, years	1347
Median (range)	37 (18–75)
18–19	5 (0.4%)
20–29	259 (19.2%)
30–39	508 (37.7%)
40–49	326 (24.2%)
≥50	249 (18.5%)
Age at GCT diagnosis, years	1342
Median (range)	31(15–61)
15–19	70 (5.2%)
20–29	497 (37%)
30–39	470 (35%)
40–49	239 (17.8%)
≥50	66 (4.9%)
Site of GCT	1342
Testis	1202 (89.6%)
Extragonadal	137 (10.2%)
Both^a^	3 (0.2%)
Histology	1330
Nonseminoma/mixed GCT	952 (71.6%)
Seminoma	372 (28%)
Both^b^	6 (0.5%)
Clinical stage at diagnosis	1190
I	378 (31.8%)
II	513 (43.1%)
III^c^	299 (25.1%)
Cumulative dose of cisplatin (mg/m^2^)	1334
Median (range)	400 (100–1000)
<300	60 (4.5%)
301–399	551 (41.3%)
400	674 (50.5%)
>400	49 (3.7%)
Ethnicity	1328
White	1093 (82.3%)
Asian	63 (4.7%)
Hispanic	20 (1.5%)
Black or African American	11 (0.8%)
Other^d^	141 (10.6%)
Education	1334
High school or less	131 (9.8%)
After high school but not college^e^	280 (21%)
College/university graduate	583 (43.7%)
Postgraduate level	327 (24.5%)
Other/prefer not to say/not answered	13 (1%)
Employment status	1315
Employed	1170 (89%)
Unemployed	89 (6.8%)
Retired	25 (1.9%)
On disability leave	31 (2.4%)
Marital status	1319
Married/living as married	803 (60.9%)
Single or never married	441 (33.4%)
Widowed, divorced, separated	75 (5.7%)

^a^ Subjects with more than one entry in their medical records that matched both tumor sites.

^b^ Subjects with more than one entry in their medical records that matched both histological categories.

^c^ Includes clinical stage IIIC (*n* = 50).

^d^ Other ethnicities also include subjects who identified with two or more ethnicities.

^e^ Subjects who answered “some college/university” education, but did not specify graduation are placed in this group.

### 3.1 Age, cumulative dose of cisplatin, and self‐reported health

We observed a significant association between age at audiometry and SRT‐based hearing disability (odds ratio (OR), 1.09; 95% confidence interval (CI), 1.07–1.11; *p* < 0.001; Figure 2A). Age was used as a covariate in the proceeding analyses. Unlike age, we did not observe a significant relationship between administered cumulative dose of cisplatin during cancer treatment and SRT‐based hearing disability (*p =* 0.72; Figure 2B). SRT‐based hearing disability was significantly associated with self‐reported health, and subjects with SRT‐based hearing disability had higher odds of reporting poor health (OR, 1.69; 95% CI, 1.18–2.42; *p =* 0.004; Figure 2C).

### 3.2 Tinnitus and “self‐reported hearing loss”

Subjects who reported hearing loss or ‘problem hearing in crowds’ had significantly higher odds of having SRT‐based hearing disability (“self‐reported hearing loss”: OR, 8.75; 95% CI, 4.92–15.55; *p* < 0.001; “problem hearing in crowds”: OR, 2.68; 95% CI, 1.77–4.06; *p* < 0.001; Figure 3A,B). There was also a significant association between tinnitus and SRT‐based hearing disability (OR, 3.60; 95% CI, 2.22–5.82; *p* < 0.001; Figure 3C).

### 3.3 Peripheral neuropathies

Significant associations with SRT‐based hearing disability were observed for peripheral motor neuropathy (OR, 3.80; 95% CI, 1.48–8.95; *p =* 0.003; Figure 4A). We did not observe a significant relationship between peripheral sensory neuropathy and SRT‐based hearing disability (*p* = 0.23; Figure 4B).

### 3.4 Smoking, heavy drinking, BMI, and physical activity

With regards to years of smoking, 431 of 477 subjects who answered “yes” to ever smoking cigarettes (Figure S4B) also provided the number of years.

Years of smoking were significantly associated with SRT‐based hearing disability (OR, 1.04; 95% CI, 1.01–1.08; *p* = 0.02; Figure 5A). Current smoking was also significantly associated with SRT‐based hearing disability (OR, 2.89; 95% CI, 1.45–5.46; *p* = 0.002; Figure 5B). We did not observe a significant relationship between heavy drinking and SRT‐based hearing disability (*p* = 0.33; Figure 5C). On the other hand, the odds of SRT‐based hearing disability increased with higher BMI (OR, 1.07; 95% CI, 1.03–1.10; *p* < 0.001; Figure 5D). There was a significant inverse relationship between physical activity and SRT‐based hearing disability with higher physical activity associated with lower odds of SRT‐based hearing disability (OR, 0.989; 95% CI, 0.980–0.996; *p* = 0.005; Figure 5E).

### 3.5 Hypertension and hypercholesterolemia

We did not observe an association with SRT‐based hearing disability for hypertension (*p* = 0.68; Figure S5C) or hypercholesterolemia (*p* = 0.95; Figure S5D).

### 3.6 Genome‐wide association

**FIGURE 6:**
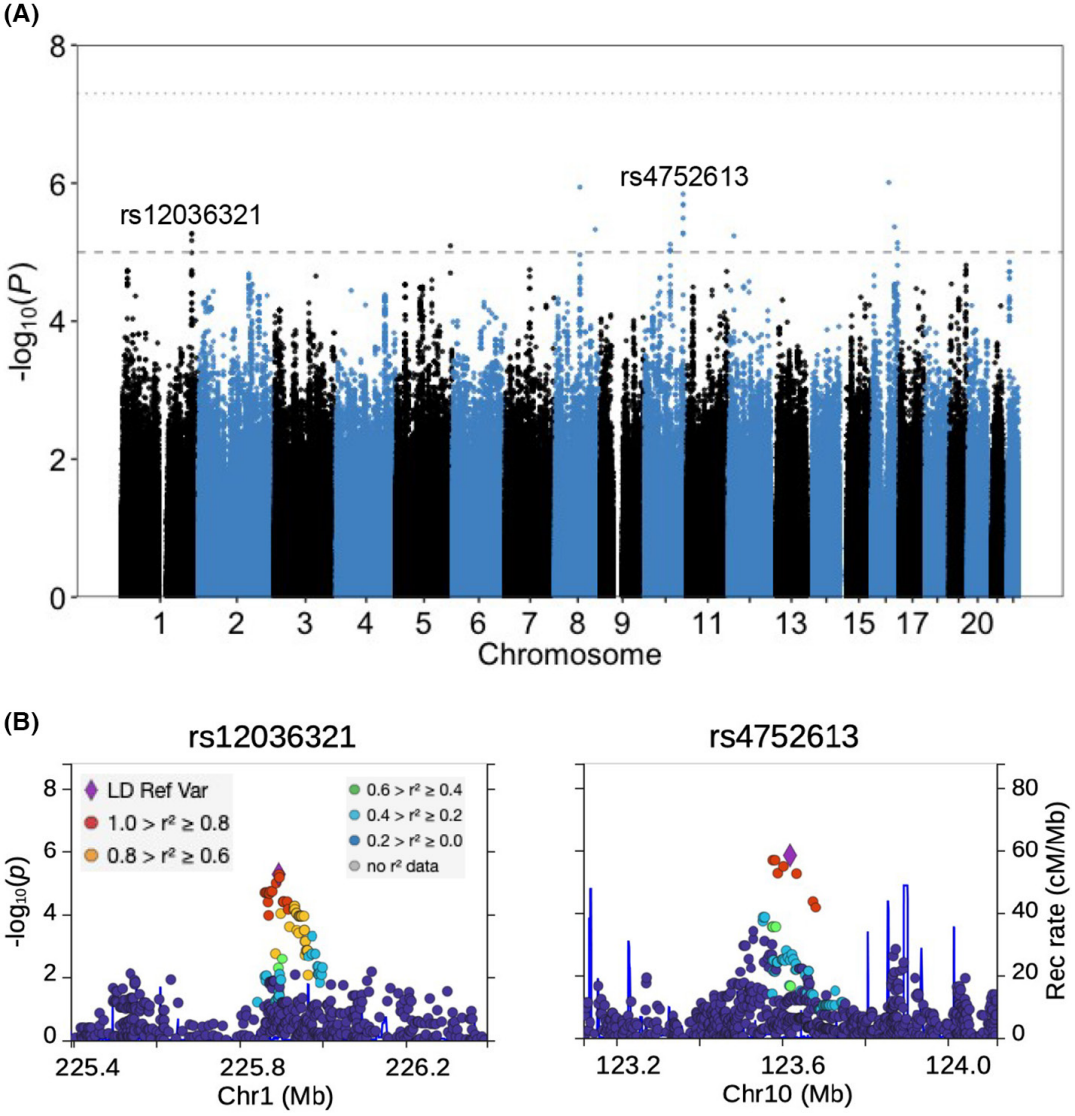
(A) Manhattan plot of genome‐wide analysis. (B) Regional Manhattan plots at the vicinity of rs12036321 and rs4752613. In (A), dashed and dotted lines are 10^−5^ and 5 × 10^−8^ thresholds, respectively

Our genome‐wide association study (GWAS) did not identify any variants with *p* value smaller than the genome‐wide significance threshold of 5 × 10^−8^; however, we identified 22 variants from 10 linkage disequilibrium (LD) blocks with *p* values lower than 10^−5^, a suggestive threshold for genome‐wide associations in humans (Figure 6A). LocusZoom was used to plot the local Manhattan plots for the most significant SNP within each LD block (Figure 6B and Figure S10). Among the most significant SNPs in each of the 10 LD blocks, three were intronic variants for non‐coding RNA genes, two (rs4752613 and rs2173537) were intronic variants for protein coding genes and one (rs2228200) was a synonymous variant for a protein coding RNA. Furthermore, three of these 10 SNPs had identified eQTL roles with one (rs12036321) also a sQTL (Table S2). The distribution of rank‐based normal transformed SRT across genotypes, as well as distribution of residuals from the regression model for two of the target SNPs, (rs12036321 and rs4752613) are shown in Figure S11.

## 4 DISCUSSION

We studied a well‐established cohort of cisplatin‐treated testicular cancer survivors to investigate risk factors for impaired speech recognition threshold. As shown previously in the general population,^12^, ^13^ we observed a strong correlation between SRT and PTA at 0.5, 1, and 2 kHz, in cancer survivors. Factors with significant associations with SRT‐based hearing disability were age, tinnitus, self‐reported hearing loss, peripheral motor neuropathy, current smoking, years of smoking, BMI, and physical activity. SRT‐based hearing disability was associated with lower self‐reported health. No association with SRT‐based hearing disability was found for cumulative dose of cisplatin, hypertension, heavy drinking, hypercholesterolemia, and peripheral sensory neuropathy. Through genome‐wide association analysis, we identified 22 genetic variants from 10 LD blocks with suggestive association with SRT decline; three of these top SNPs are eQTLs or are within coding region of genes (*ATE1*, *ZFHX3, ENAH*, and *SRP9*) that play important roles in neural development and function.

### 4.1 Age, cumulative dose of cisplatin, and self‐reported health

Consistent with age‐related hearing declines, we observed a significant association between SRT‐based hearing disability and age. Cumulative dose of cisplatin was not associated with SRT‐based hearing disability, suggesting the possibility that SRT deterioration is independent of the ototoxic effect of cisplatin. SRT is highly correlated with low frequency hearing sensitivity measured by PTA of 0.5, 1, and 2 kHz. Thus, our lack of association between SRT and cisplatin dose is consistent with results by others demonstrating cisplatin‐induced hearing loss in the high frequency range,^15^, ^16^, ^17^, ^18^ and our own results showing significant associations between dose of cisplatin and hearing loss at 4, 6, 8, 10, and 12 kHz, but not at lower frequencies (0.25, 0.5, 1, 2, 3 kHz).^2^ Hearing loss directly impacts speech recognition. In a previous study,^48^ speech perception was measured in quiet and with background noise three decades after cisplatin treatment, and results indicated no difference between cases and age‐matched controls consistent with our SRT data measured within a few years of cisplatin treatment.

The observed association between self‐reported health and SRT‐based hearing disability could indicate an impact of hearing disability on the subjects' perception of health. However, association does not imply causation, and other underlying conditions influencing health perception could contribute to SRT deterioration and, therefore, to the observed association.

### 4.2 Tinnitus and “self‐reported hearing loss”

We have previously reported associations of both tinnitus and “self‐reported hearing loss” with SRT deterioration in a subset of The Platinum Study cohort^2^ which we now confirm in an expanded dataset. Our observed associations of ‘self‐reported hearing loss’ and ‘problem hearing in crowds’ with SRT‐based hearing disability are expected. Hearing loss directly impacts speech recognition. Conversely, difficulty in hearing everyday speech is one of the primary means of recognizing hearing loss. In a longitudinal study of testicular cancer survivors, using self‐report for hearing loss and tinnitus, both conditions were 3–4 times more common in survivors 30 years post‐treatment compared with controls (hearing loss: 23% cases/7% controls, *p* = 0.007; tinnitus: 38% cases/10% controls, *p* < 0.001).^48^ The observed association of tinnitus with SRT‐based hearing disability might be due to informational masking of tinnitus on speech recognition^49^ or due to potential shared causal mechanisms.

### 4.3 Smoking, drinking, BMI, and physical activity

A 2005 meta‐analysis concluded a suggestive positive association between smoking and hearing loss.^50^ History of smoking was also associated with self‐reported hearing loss^51^ and deterioration of SRT in noise was significantly more rapid among adults with a history of smoking in a 10‐year longitudinal study.^52^ In addition, long‐term smokers with occupational noise exposure were found to have a higher risk of developing permanent hearing loss at 3 and 4 kHz when compared with non‐smokers with a similar occupational history.^53^ Current smoking was also significantly associated with hearing loss at low frequency PTA (0.5, 1, 2, 4 kHz).^54^ Here we report associations of both current smoking and years of smoking with SRT‐based hearing disability among cancer survivors, highlighting the potential role of smoking as a risk factor for SRT‐based hearing disability. However, it is also possible that smoking could correlate with lifestyle or behavioral factors that contribute to poor speech recognition outcomes.

We did not find a significant association between heavy alcohol consumption and SRT‐based hearing disability, consistent with the reported lack of association between decline of SRT (in noise) and alcohol use in a longitudinal study.^52^ Moderate alcohol consumption has been shown to be inversely correlated with hearing loss.^55^, ^56^ In those with a history of heavy drinking, Popelka et al.^56^ have reported a 35 percent increase in the odds of having high frequency hearing loss (PTA of 4, 6, 8 kHz); however, these frequencies have a limited overlap with the speech range studied here.

We observed significant associations between increasing BMI as well as lower physical activity with SRT‐based hearing disability. Associations between BMI or ‘obesity defined by BMI’ with hearing loss have been reported in both cross sectional^55^, ^57^ and longitudinal studies.^58^ Higher BMI and low physical activity have been associated with greater hearing loss in women.^59^ Low physical activity was also associated with hearing loss based on low frequency PTA (0.5, 1, 2, 4 kHz).^60^ Cross‐sectional association of obesity with higher SRT in noise has also been reported despite lack of a longitudinal association.^61^ These reports alongside our findings suggest that obesity and lack of physical activity could contribute to SRT deterioration. Alternatively, declines in auditory processing could impact the ability of survivors to perform physical activity or there could be shared causal mechanisms including lifestyle factors that contribute to the observed association.

### 4.4 Peripheral neuropathies

We did not observe a significant association between peripheral sensory neuropathy and SRT‐based hearing disability. However, there was a significant association between peripheral motor neuropathy and SRT‐based hearing disability. There is a wide array of identified factors that disproportionately contribute to the development of either peripheral sensory neuropathy or peripheral motor neuropathy.^62^, ^63^, ^64^ The difference in the outcome of association analyses of sensory or motor neuropathies with SRT‐based hearing disability that we observed could be due to differences in these underlying factors. For instance, potential causal factors could contribute to both motor neuropathy and SRT decline without affecting sensory neuropathy. Conversely, potential causal factors for sensory neuropathy may not influence either SRT or motor neuropathy. Further, consistent with the intricate interaction of auditory and motor pathways,^65^ it is also plausible that auditory decline could negatively affect motor performance, leading to poor peripheral motor function or self‐assessment of it.

### 4.5 Hypertension and hypercholesterolemia

We did not observe an association of hypertension or hypercholesterolemia (defined by taking prescription medications) with SRT‐based hearing disability. There are conflicting reports about the association of hypertension and hearing loss, with presence or lack of association being reported.^57^, ^66^ Longitudinal studies have failed to show associations between hypertension and hearing loss.^55^, ^58^ Interestingly, Gates et al. have reported hypertension in men to be correlated with PTA at higher frequencies (4, 6, 8 kHz), but not at PTA of lower frequencies (0.25, 0.5, 1 kHz).^67^ A recent report showed a general association of hypertension with SRT in noise, but did not find a longitudinal association.^61^ Differences in study designs and target populations might account for these seemingly contradictory reports.

### 4.6 Genome‐wide association study

Suggestively implicated variants were assessed for location (coding, intronic, intergenic) and we used the GTEx portal to check whether the leading variants in each LD block were eQTLs or sQTLs. rs4752613 is located within the intronic sequence of *ATE1* and is an eQTL for it. ATE1 regulated neurite formation during development of the brain^68^ and noticeable ATE1 levels have also been detected in hair cells of mouse.^69^ rs2228200 yields synonymous variants within coding region of *ZFHX3(ATBF1)* which encodes a transcription factor involved in neuronal differentiation^70^ and in protection of cerebellar neurons from oxidative stress.^71^ The chicken ortholog of *ZFHX3* is expressed in otic placode^72^ and the expression of *ZFHX3* is known to be regulated during development of inner ear in mouse.^73^The SNP, rs12036321, is an eQTL for *ENAH*, a gene that is highly expressed in the nervous system and encodes a protein that regulates local translation in developing axons.^74^ rs12036321 is also an eQTL for *SRP9* and a protein heterodimer that includes SRP9 potentially regulates protein biosynthesis in dendrites.^75^, ^76^ In addition, rs12036321 is an sQTL for *TMEM63A* which is expressed in oligodendrocytes and heterozygous missenses mutations in *TMEM63A* lead to transient hypomyelination during infancy.^77^, ^78^

We also compared our suggestive SNPs with the list of 51 identified SNPs associated with age‐related hearing loss.^79^ Only rs1702244 was within 100 kb (82 kb downstream) of a reported SNP (rs12784122). rs1702244 is also 86 kb downstream of *SH2D4B* gene which has a noticeable expression in mouse hair cells.^79^ The limited overlap between the two SNP sets is not surprising, considering that our study subjects are primarily young male cancer survivors. This demography is expected to restrict our dataset's ability in identification of genetic components of age‐related hearing loss.

### 4.7 Study limitations

A potential limitation for the impact and generalization of our findings to other populations is that 82.3% of the subjects in clinical associations and all of the subjects in genome‐wide association are white.

Another limitation of the current study is reliance on questionnaire‐based evaluation of most of the clinical variables. Some of these variables could intrinsically be obtained through self‐assessment (self‐reported health, tinnitus, problem hearing in the crowds). Other life style variables such as smoking and drinking habits are usually self‐reported considering the limitations of real‐life tracking of these behaviors. Biases are intrinsic limitations of cross sectional studies.^80^ The recall bias in particular might affect the control groups for smoking, hypertension, and hypercholesterolemia who answered never smoking or taking the medications or when the total years of smoking were reported. Nevertheless, the questions used for the study mainly evaluate current or immediate past experiences of the subjects which are expected to be less susceptible to recall bias.

Lack of information on other ototoxic drugs (i.e., aminoglycosides) is another limitation of the study; however, we do not expect a systematic exposure of the participants (primarily young adults) to other major ototoxic medication.

Lack of identification of SNPs reaching significance cutoff of 5 × 10^−8^ and lack of a replication dataset are limitations of our genome‐wide association study. To the best of our knowledge, there are no datasets currently available that would allow for an accurate replication of our genome‐wide associations. Emergence of new datasets and increased sample size would provide the means for expansion of the current findings on the genetic architecture of SRT decline in cancer survivors.

### 4.8 Summary

Our results improve the current understanding of risk factors for the decline of speech recognition in cancer survivors. Higher BMI, lower physical activity, and smoking are associated with SRT decline.

Image Integrity: LocusZoom images are modified to increase legibility and to remove redundant annotations.

## AUTHOR CONTRIBUTIONS

**Mohammad Shahbazi:** Conceptualization (lead); formal analysis (lead); investigation (equal); validation (lead); writing – original draft (lead); writing – review and editing (equal). **Xindi Zhang:** Conceptualization (equal); investigation (equal); methodology (equal); writing – review and editing (equal). **Paul C Dinh:** Data curation (equal); investigation (equal); methodology (equal); writing – review and editing (equal). **Victoria A Sanchez:** Conceptualization (equal); investigation (equal); writing – review and editing (equal). **Matthew R Trendowski:** Investigation (equal); methodology (equal); writing – review and editing (equal). **Megan Shuey:** Formal analysis (equal); investigation (equal); writing – review and editing (equal). **Tessa Nguyen:** Investigation (equal); writing – review and editing (equal). **Darren R. Feldman:** Investigation (equal); writing – review and editing (equal). **David J. Vaughn:** Investigation (equal); writing – review and editing (equal). **Chunkit Fung:** Investigation (equal); writing – review and editing (equal). **Christian K Kollmannsberger:** Investigation (equal); writing – review and editing (equal). **Neil E. Martin:** Investigation (equal); writing – review and editing (equal). **Lawrence H. Einhorn:** Investigation (equal); writing – review and editing (equal). **Nancy J Cox:** Investigation (equal); methodology (equal); writing – review and editing (equal). **Robert D Frisina:** Conceptualization (equal); investigation (equal); writing – review and editing (equal). **Lois B Travis:** Conceptualization (equal); funding acquisition (equal); investigation (equal); project administration (equal); resources (equal); supervision (equal); writing – review and editing (equal). **Mary Eileen Dolan:** Conceptualization (equal); investigation (equal); project administration (equal); writing – original draft (equal); writing – review and editing (equal).

## CONFLICT OF INTEREST

Darren R. Feldman has a relationship with Gilead, Decibel, Astellas, Seattle Genetics, Novartis, and UpToDate. Victoria A. Sanchez reports paid research support and/or consultancy stipends from Autifony Therapeutics Ltd., Oticon Medical, Otonomy Inc., and Boehringer Ingelheim International GmbH (Unrelated to the project). All other authors declare no conflicts of interest.

## ETHICAL APPROVAL STATEMENT

The study was conducted in line with the U.S. Common Rule and was approved by respective Institutional Review Boards. All subjects were >18 years old during enrollment and provided written consent for participation including access to medical records and genetic analysis.

## Supporting information


Appendix S1

## Data Availability

For summary statistics of the GWAS of Speech Recognition Threshold presented in this study, see: https://apps.cancer.iu.edu/platinum/published‐research.php
