# Supplementary material for: Comprehensive association analysis of speech recognition thresholds after cisplatin‐based chemotherapy in survivors of adult‐onset cancer
Source: Cancer Med. 2022 Sep 12;12(3):2999–3012. doi: 10.1002/cam4.5218 (PMC9939144; doi:10.1002/cam4.5218)
Supplement: Supplementary file 1 — Appendix S1 [file CAM4-12-2999-s001.pdf]

Supplemental Material for:

Comprehensive Association Analysis of Speech Recognition Thresholds after Cisplatin-based Chemotherapy in Survivors of Adult-onset Cancer

Mohammad Shahbazi[1], Xindi Zhang[1], Paul Dinh[2], Victoria A. Sanchez[3], Matthew R. Trendowski[1], Megan M. Shuey[4], Tessa Nguyen[5], Regeneron Genetics Center[*], Darren R. Feldman[6], David J. Vaughn[7], Chunkit Fung[8], Christian Kollmannsberger[9], Neil E. Martin[10], Lawrence H. Einhorn[2], Nancy J. Cox[4], Robert D. Frisina[11], Lois B. Travis[2] and M. Eileen Dolan[1]

[1] Department of Medicine, University of Chicago, Chicago, IL, USA;
[2] Department of Medical Oncology, Indiana University, Indianapolis, IN;
[3] Department of Otolaryngology – Head and Neck Surgery, University of South Florida, Tampa, FL, USA;
[4] Department of Medicine and Vanderbilt Genetics Institute, Vanderbilt University Medical Center, Nashville, TN;
[5] Center for Audiology, Speech, Language and Learning, Northwesthern University, Chicago, IL;
[*]Regeneron Genetics Center (RGC) authors/contributors are listed in alphabetical order.

RGC Management and Leadership Team: Goncalo Abecasis, Ph.D., Aris Baras, M.D., Michael Cantor, M.D., Giovanni Coppola, M.D., Aris Economides, Ph.D., Luca A. Lotta, M.D., Ph.D., John D. Overton, Ph.D., Jeffrey G. Reid, Ph.D., Alan Shuldiner, M.D.
Contribution: All authors contributed to securing funding, study design and oversight. All authors reviewed the final version of the manuscript.

Sequencing and Lab Operations: Christina Beechert, Caitlin Forsythe, M.S., Erin D. Fuller, Zhenhua Gu, M.S., Michael Lattari, Alexander Lopez, M.S., John D. Overton, Ph.D., Thomas D. Schleicher, M.S., Maria Sotiropoulos Padilla, M.S., Louis Widom, Sarah E. Wolf, M.S., Manasi Pradhan, M.S., Kia Manoochehri, Ricardo H. Ulloa.
Contribution: C.B., C.F., A.L., and J.D.O. performed and are responsible for sample genotyping. C.B, C.F., E.D.F., M.L., M.S.P., L.W., S.E.W., A.L., and J.D.O. performed and are responsible for exome sequencing. T.D.S., Z.G., A.L., and J.D.O. conceived and are responsible for laboratory automation. M.P., K.M., R.U., and J.D.O are responsible for sample tracking and the library information management system.

Genome Informatics: Xiaodong Bai, Ph.D., Suganthi Balasubramanian, Ph.D., Andrew Blumenfeld, Boris Boutkov, Ph.D., Gisu Eom, Lukas Habegger, Ph.D., Alicia Hawes, B.S., Shareef Khalid, Olga Krasheninina, M.S., Rouel Lanche, Adam J. Mansfield, B.A., Evan K. Maxwell, Ph.D., Mrunali Nafde, Sean O'Keeffe, M.S., Max Orelus, Razvan Panea, Ph.D., Tommy Polanco, B.A., Ayesha Rasool, M.S., Jeffrey G. Reid, Ph.D., William Salerno, Ph.D., Jeffrey C. Staples, Ph.D.

Contribution: X.B., A.H., O.K., A.M., S.O., R.P., T.P., A.R., W.S. and J.G.R. performed and are responsible for the compute logistics, analysis and infrastructure needed to produce exome and genotype data. G.E., M.O., M.N. and J.G.R. provided compute infrastructure development and operational support. S.B., S.K., and J.G.R. provide variant and gene annotations and their functional interpretation of variants. E.M., J.S., R.L., B.B., A.B., L.H., J.G.R. conceived and are responsible for

creating, developing, and deploying analysis platforms and computational methods for analyzing genomic data.

Research Program Management: Marcus B. Jones, Ph.D., Jason Mighty, Ph.D., Lyndon J. Mitnaul, Ph.D.

Contribution: All authors contributed to the management and coordination of all research activities, planning and execution. All authors contributed to the review process for the final version of the manuscript;

[6] Department of Medical Oncology, Memorial Sloan-Kettering Cancer Center, New York, USA, NY;
[7] Department of Medicine, University of Pennsylvania, Philadelphia, PA, USA;
[8] J.P. Wilmot Cancer Institute, University of Rochester Medical Center, Rochester, NY;
[9] Division of Medical Oncology, University of British Columbia, Vancouver, BC, Canada;
[10] Department of Radiation Oncology, Dana-Farber Cancer Institute, Boston, MA;
[11] Departments of Medical Engineering and Communication Sciences and Disorders, Global Center for Hearing and Speech Research, University of South Florida, Tampa, FL, USA

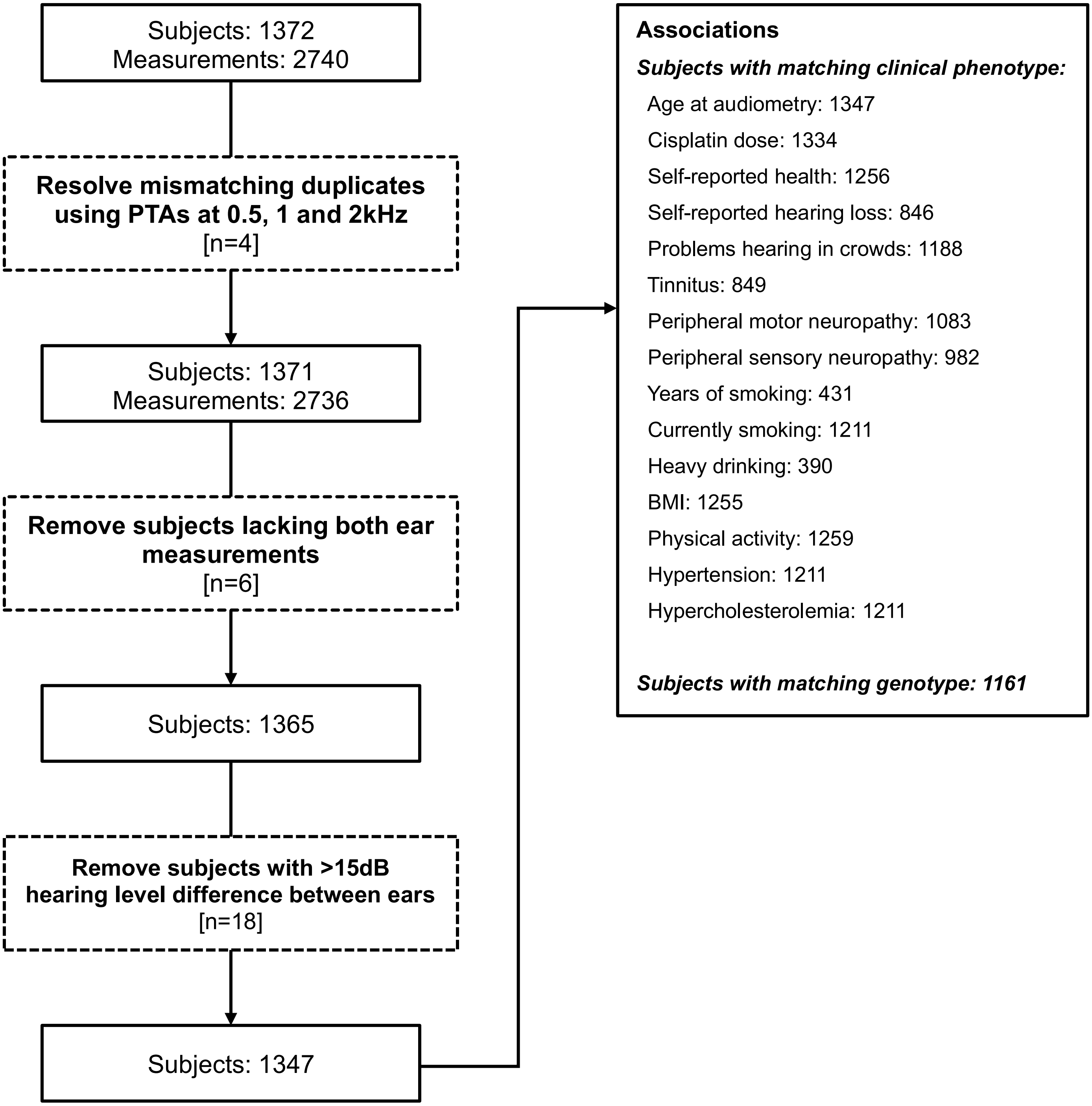

Supplemental Figure 1. Quality control of SRT measurements. The filtration steps are shown as boxes with dashed outlines and n is the number of measurements or subjects excluded.

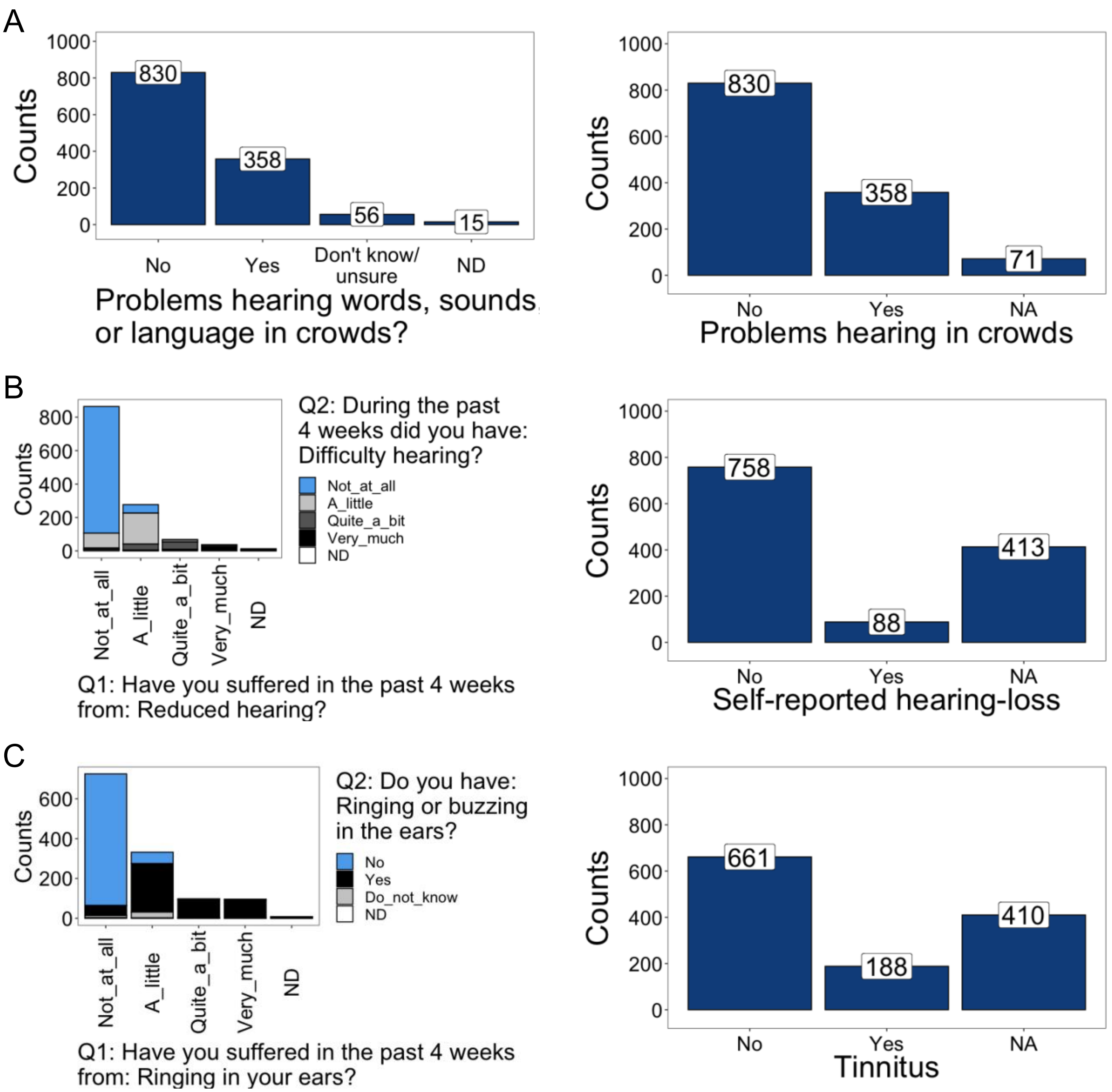

Supplemental Figure 2. Distribution of responses to questions regarding (A) problems hearing in crowds, (B) self-reported hearing loss and (C) tinnitus. The left panels show the distribution of responses to questions regarding the clinical variable and the right panels show the distribution of binary transformed data used for the association analyses with “Yes” and “No” representing case and control groups respectively. In (A), subjects that answered “Don’t know/not sure” or were without data (ND) were excluded. In (B), subjects that answered “Quite a bit” or “Very much” to both questions were placed in the hearing loss group and subjects that answered “Not at all” to both questions were placed in the control group. In (C), subjects that answered “Quite a bit” or “Very much” to question 1 and “Yes” to question 2 were placed in the tinnitus group and subjects that answered “Not at all” to question 1 and “No” to question 2 were placed in the control group.

	Question	Condition category
	**During the past 4 weeks did you have:**	
a	Tingling fingers or hands?	Sensory
b	Tingling toes or feet?	Sensory
c	Numbness in your fingers or hands?	Sensory
d	Numbness in your toes or feet?	Sensory
e	Shooting or burning pain in your fingers or hands?	Sensory
f	Shooting or burning pain in your toes or feet?	Sensory
g	Cramps in your hands?	Motor
h	Cramps in your feet?	Motor
i	Problems standing or walking because of difficulty feeling the ground under your feet?	Sensory
j	Difficulty in distinguishing between hot and cold water?	Sensory
k	Problems holding a pen which made writing difficult?	Motor
l	Difficulty manipulating small objects with your fingers (for example, fastening small buttons)?	Motor
m	Difficulty opening a jar or bottle because of weakness in your hands?	Motor
n	Difficulty walking because your feet dropped downwards?	Motor
o	Difficulty in climbing stairs or getting up out of a chair because of weakness in your legs?	Motor
p	Dizzy when standing up from a sitting or lying position?	Autonomic [Excluded]
q	Blurred vision?	Autonomic [Excluded]
r	Difficulty hearing?	Sensory [Excluded]
s	Please respond only if you drive a car: difficulty using the pedals?	Motor [Excluded]
t	Getting or maintaining an erection?	Autonomic [Excluded]

Supplemental Table 1. European organization for research and treatment of cancer (EORTC) questionnaire for chemotherapy-induced peripheral neuropathy (CIPN20). Each question could be answered with “Not at all”, “A little”, “Quite a bit” and “Very much”. Question ‘r’ was used as a part of self-reported hearing loss evaluation presented in Supplemental Fig. 1B. Question ‘s’ was excluded as it was restricted to driving subjects. Peripheral autonomic neuropathy was excluded since question ‘t’ is expected to be affected by the history of germline tumor in subjects and the remaining two questions (‘p’ and ‘q’) might not be sufficient for the evaluation of autonomic neuropathy. Sensory, peripheral sensory neuropathy; motor, peripheral motor neuropathy; autonomic, peripheral autonomic neuropathy.

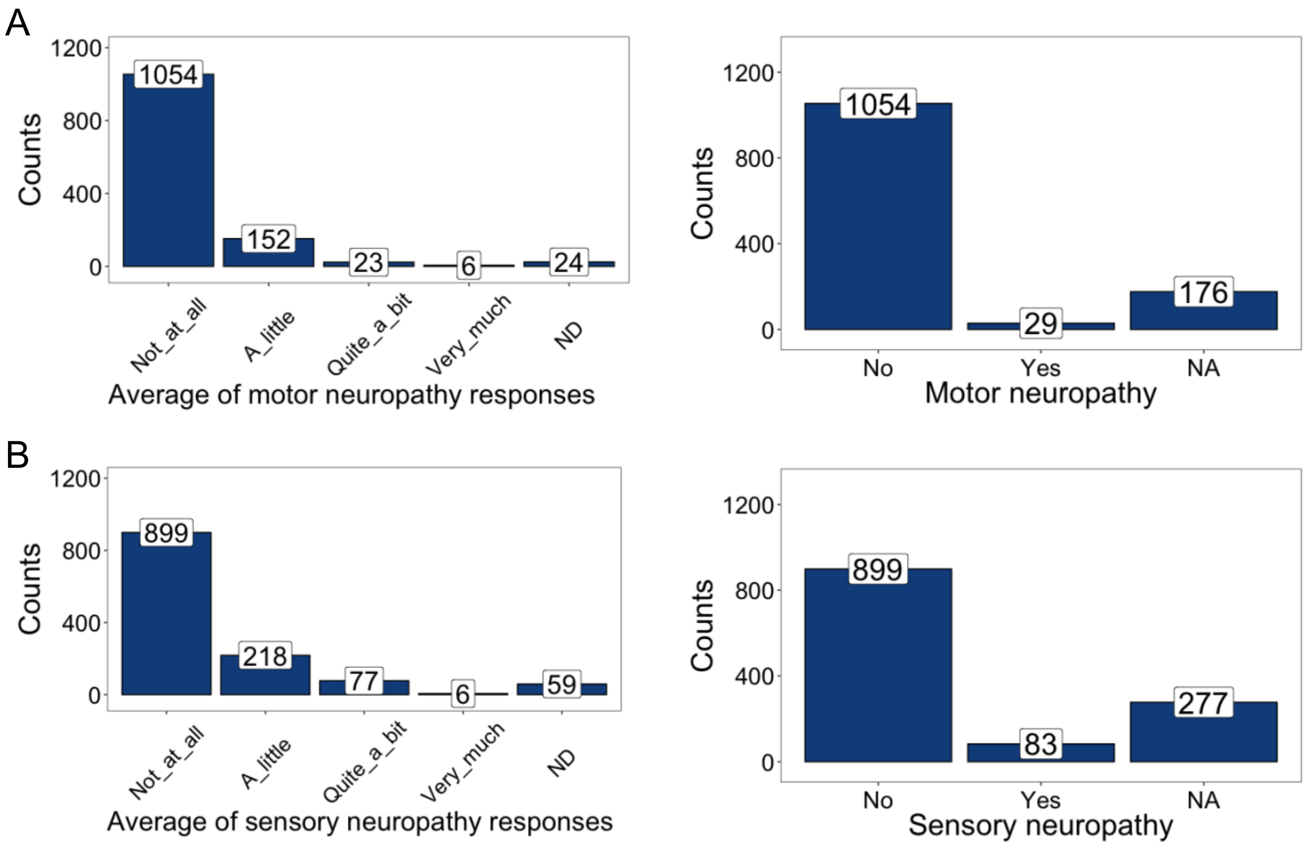

Supplemental Figure 3. Distribution of responses to questions regarding (A) peripheral motor neuropathy and (B) peripheral sensory neuropathy. The left panels show the distribution of responses to questions regarding the clinical variable and the right panels show the distribution of binary transformed data used for the association analyses with “Yes” and “No” representing case and control groups respectively. Subjects that answered “Quite a bit” or “Very much” were placed in the neuropathy group and subjects that answered “Not at all” were placed in the control group.

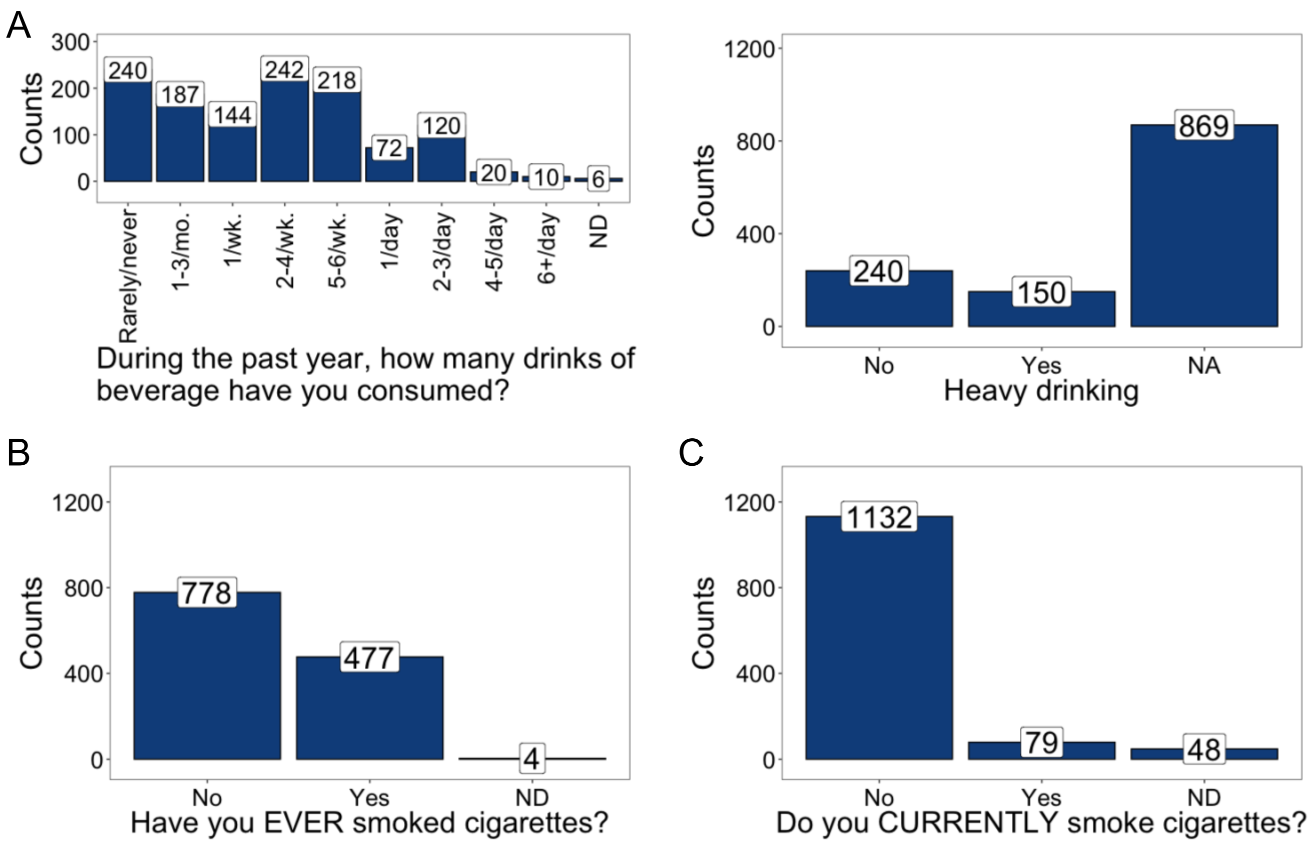

Supplemental Figure 4. Distribution and binary transformation of responses to questions regarding (A) heavy drinking, (B) ever smoking and (C) current smoking. In (A), the left panel shows the distribution of responses to questions regarding heavy drinking and the right panel shows the distribution of binary transformed data used for the association analysis with “Yes” and “No” representing case and control groups respectively. Subjects that answered “2-3/day”,”4-5/day” and “6+/day” were placed in the heavy drinking group and subjects that answered “Rarely/never” were placed in the control group. In (B) subjects that answered “Yes” were evaluated for “years of smoking”.

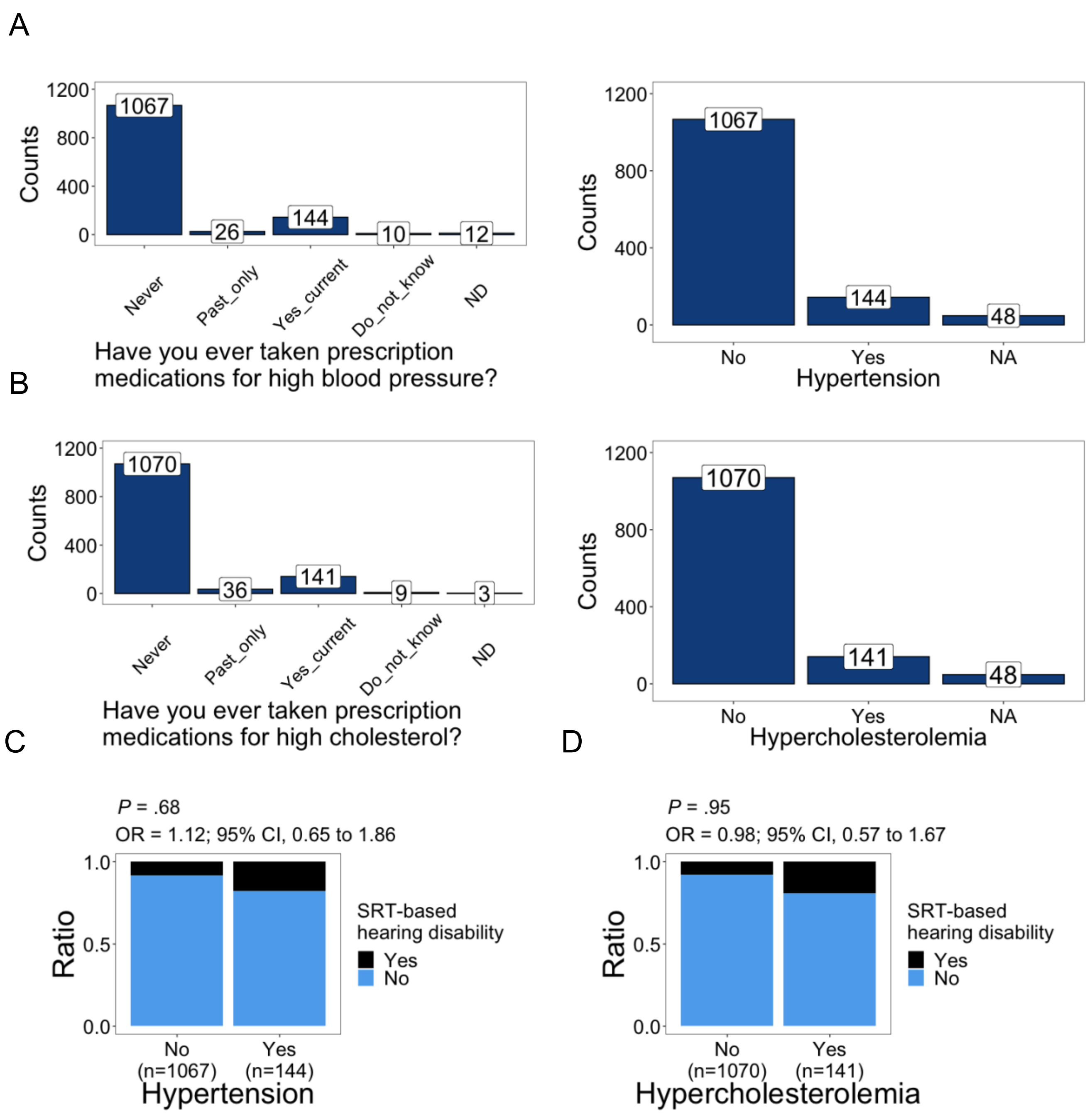

Supplemental Figure 5. Distribution of hypertension and hypercholesterolemia data and their association with SRT-based hearing disability. In (A, B), The left panels show the distribution of responses to questions regarding the clinical variable and the right panels show the distribution of binary transformed data used for the association analyses with "Yes" and "No" representing case and control groups respectively. Subjects that answered "Yes_current" were placed in the condition group and subjects that answered "Never" were placed in the control group. (C, D) show distribution of SRT-based hearing disability relative to binary transformed hypertension and hypercholesterolemia. *P* values and odds ratios (OR) are calculated using logistic regression model: hearing disability = response + age at audiometry.

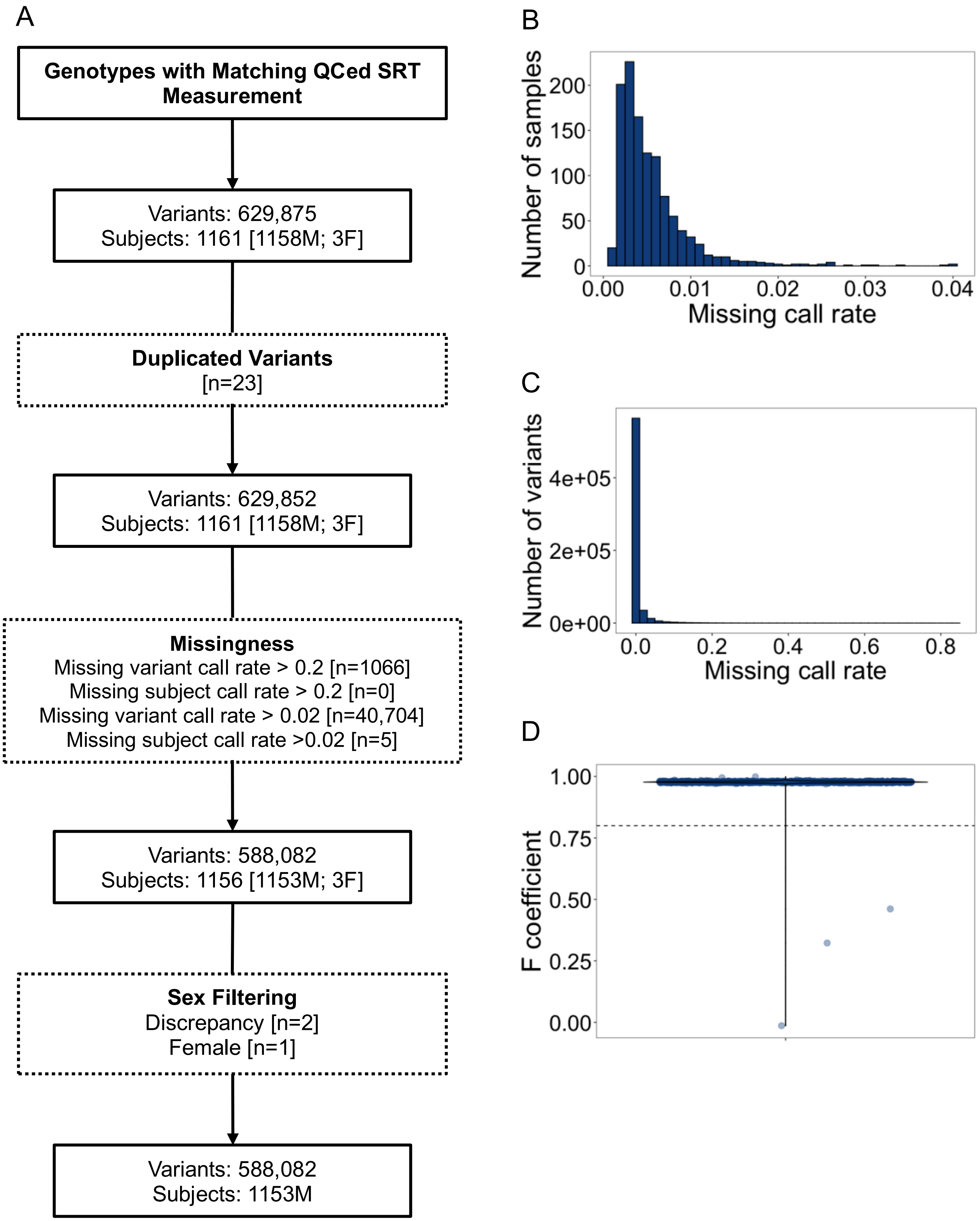

Supplemental Figure 6. Quality control of genotype data based on duplicated variants, missing call rates and sex. (A) Flow chart with the filtration steps as boxes with dashed lines and n as the number

of variants or subjects excluded. (B) Pre-filtration distribution of missing call rates of variants. (C) Pre-filtration distribution of missing call rates of subjects. (D) Distribution of X chromosome homozygosity rate. Dotted line at 0.8 is the threshold used for selection of male genotypes. F refers to Female; M refers to Male.

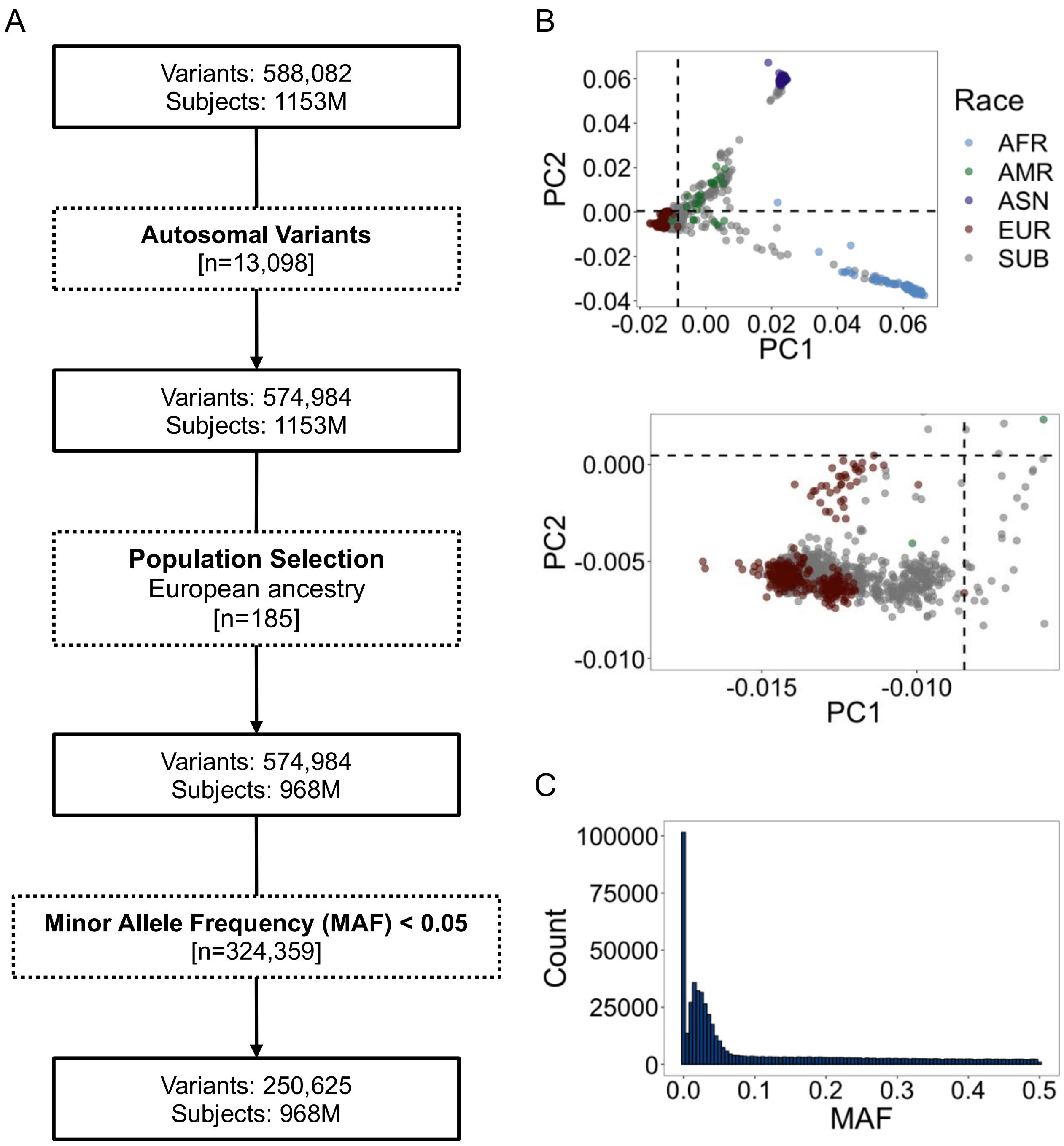

Supplemental Figure 7. Quality control of genotype data to select autosomal variants, infer ancestry of subjects and, remove variants with low minor allele frequency. (A) Flow chart with the filtration steps as boxes with dashed lines and n as the number of variants or subjects excluded. (B) Principal component plot of the genotypes anchored by 1000 genome data, for ancestry inference and population selection. The top plot shows the global distribution alongside cutoff lines. Subjects with European ancestry (the bottom left box) were selected. The lower plot shows the selected region in higher details. (C) Distribution of minor allele frequency across variants. AFR, African; AMR, Admixed American; ASN, Asian; EUR, European; SUB, study subjects. M refers to Male.

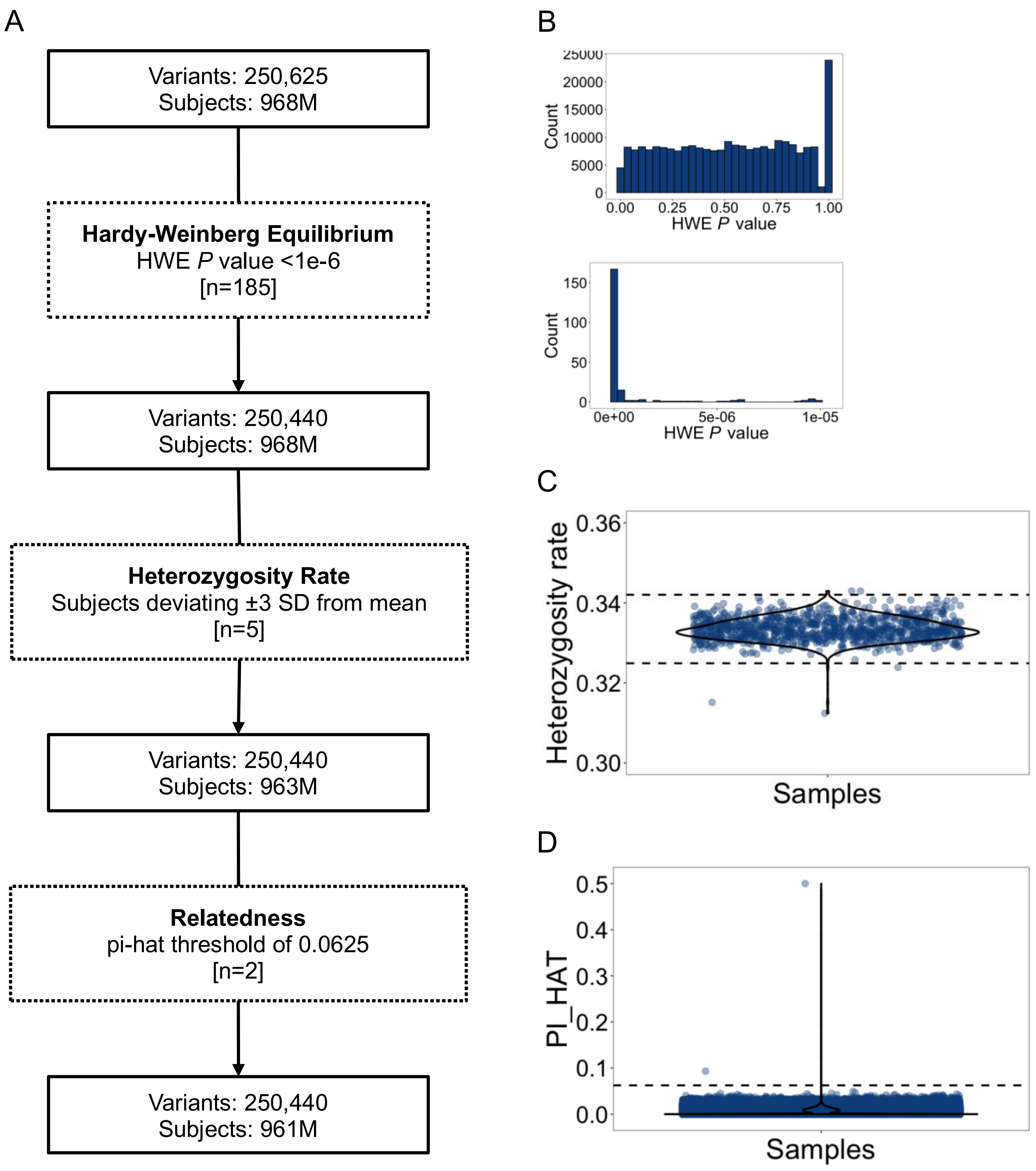

Supplemental Figure 8. Quality control of genotype data based on deviation from HWE, heterozygosity rate and relatedness. (A) Flow chart with the filtration steps as boxes with dashed lines and n as the number of variants or subjects excluded. (B) Distribution of HWE *P* value across variants. The top plot shows global distribution, and the lower plot shows the distribution at the strong deviation region of P values. (C) Heterozygosity rate of subjects alongside cutoff lines (±3 x SD). (D) Filtration of samples based on relatedness with dotted line showing the 0.0625 threshold. M refers to Male.

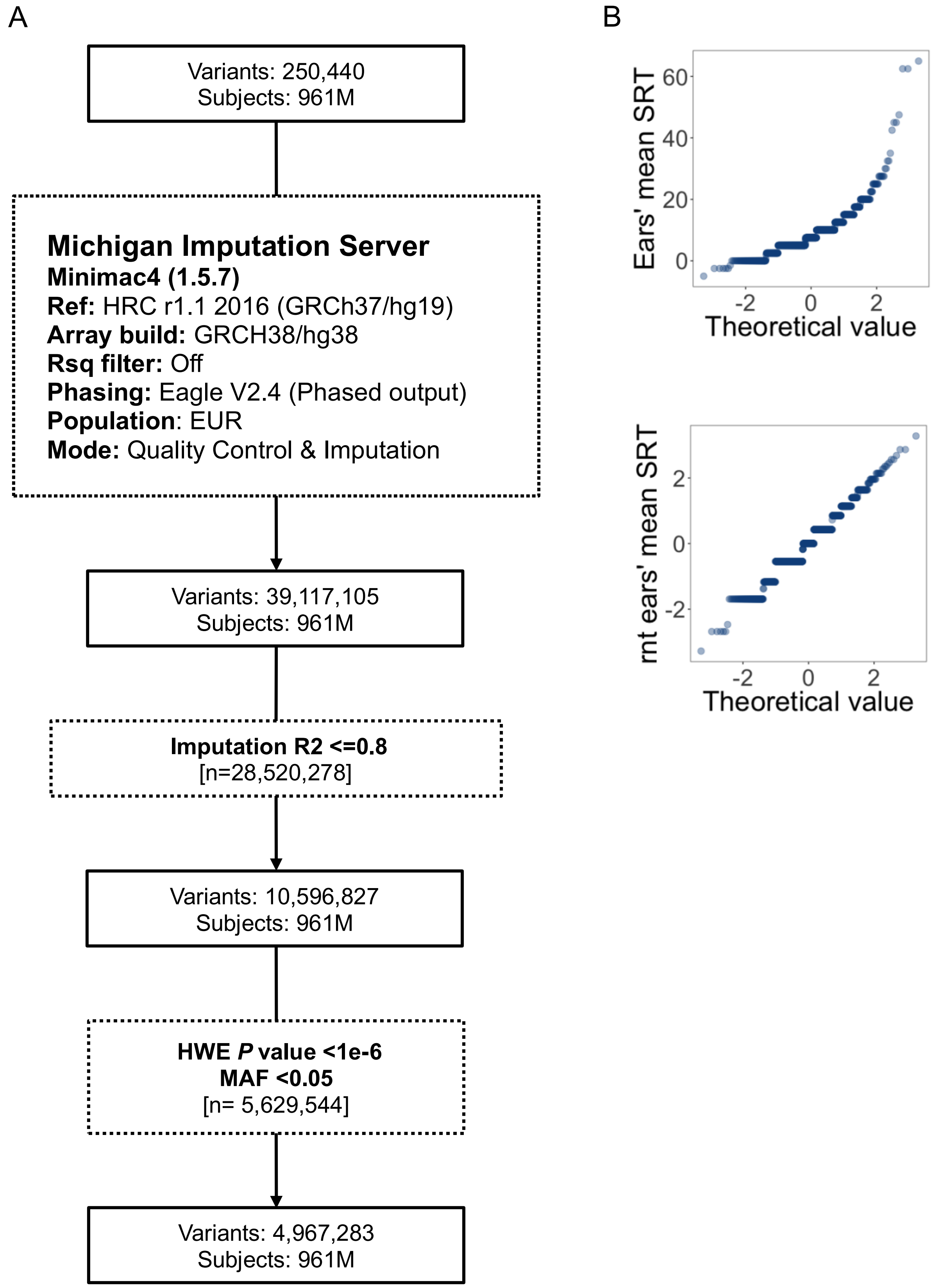

Supplemental Figure 9. Quality control of genotype data after imputation and preparation of SRT data for GWA. (A) Flow chart with the filtration steps as boxes with dashed lines and n as the number of variants or subjects excluded. (B) Rank-based inverse normal transformation of SRT data with

matching post QC genotypes for GWA. The top and the bottom quantile-quantile plots show pre and post transformation data respectively.

rsID	CHR	BP	REF	ALT	*P*	BETA	95% CI	LDB	Gene: Info	eQTL	eQTL Tissue	sQTL	sQTL Tissue
rs9928983	16	54589811	T	C	9.78E-07	-0.42	-0.58 to -0.25	1	LINC02183: Intron Variant	-	-	-	-
rs4739148	8	79846184	G	T	1.14E-06	0.25	0.15 to 0.36	2	LOC105375912: Intron Variant	-	-	-	-
									LOC105375914: Intron Variant				
rs4752613	10	123619383	G	C	1.44E-06	-0.29	-0.41 to -0.17	3	ATE1: Intron Variant	ATE1	Thyroid	-	-
											Artery		
											- Tibial		
rs11200175	10	123578232	A	C	2.05E-06	-0.29	-0.4 to -0.17	3	ATE1 : Intron Variant				
rs11200179	10	123583695	G	A	2.05E-06	-0.29	-0.4 to -0.17	3	ATE1 : Intron Variant				
rs79960024	10	123603556	T	C	3.21E-06	-0.28	-0.39 to -0.16	3	ATE1 : Intron Variant				
rs7906842	10	123590183	A	C	5.27E-06	-0.27	-0.39 to -0.16	3	ATE1 : Intron Variant				
rs111322933	10	123634964	C	T	5.45E-06	-0.27	-0.39 to -0.16	3	ATE1 : Intron Variant				
rs2228200	16	72984668	C	T	4.30E-06	-0.38	-0.54 to -0.22	4	ZFHX3: Synonymous Variant	-	-	-	-
rs2173537	8	129047325	G	A	4.70E-06	-0.24	-0.35 to -0.14	5	PVT1: Intron Variant	-	-	-	-
rs12036321	1	225894059	C	T	5.39E-06	-0.27	-0.39 to -0.15	6	LOC102723834: Intron Variant	ENAH	Spleen	TMEM63A	Adipose
											Ovary		- Subcutaneous
													Skin
													- Sun Exposed (Lower leg)
													Breast
													- Mammary Tissue
													Nerve
													- Tibial
										SRP9	Muscle	SRP9	Esophagus
											- Skeletal		- Mucosa
rs61850657	1	225895096	G	A	5.45E-06	-0.27	-0.39 to -0.15	6	LOC102723834 : Intron Variant				
rs2615061	1	225895806	G	A	6.79E-06	-0.27	-0.38 to -0.15	6	LOC102723834 : Intron Variant				
rs78093285	12	16489449	A	C	5.81E-06	-0.39	-0.55 to -0.22	7	-	RP11-239A17.1	Adipose	-	-
											- Visceral (Omentum)		
											Cells		
											- Cultured fibroblasts		
											Adipose		
											- Subcutaneous		
rs3933578	16	82622283	A	G	7.35E-06	0.19	0.11 to 0.27	8	-	-	-	-	-
rs10514545	16	82620539	G	A	8.85E-06	-0.19	-0.27 to -0.11	8					
rs1702244	10	82491835	A	G	7.69E-06	0.19	0.11 to 0.27	9	-	-	-	-	-
rs7913143	10	82480028	C	G	9.50E-06	0.19	0.1 to 0.27	9					
rs7913282	10	82480100	C	T	9.50E-06	0.19	0.1 to 0.27	9					
rs7923403	10	82480172	T	C	9.50E-06	0.19	0.1 to 0.27	9					
rs1702239	10	82489758	T	C	9.50E-06	0.19	0.1 to 0.27	9					
rs10036863	5	174990523	A	G	8.09E-06	0.21	0.12 to 0.31	10	-	-	-	-	-

Supplemental Table 2. List of SNPs with *P* values below 10^{-5} threshold. QTL data are provided for the most significant SNPs in each LD block (LDB). eQTL and sQTL information were obtained from GTEx. Information in ‘Gene: info’ column was obtained from NCBI database of genetic variation. -, no data. Alternative alleles were used as the effect alleles for the calculations in this table.

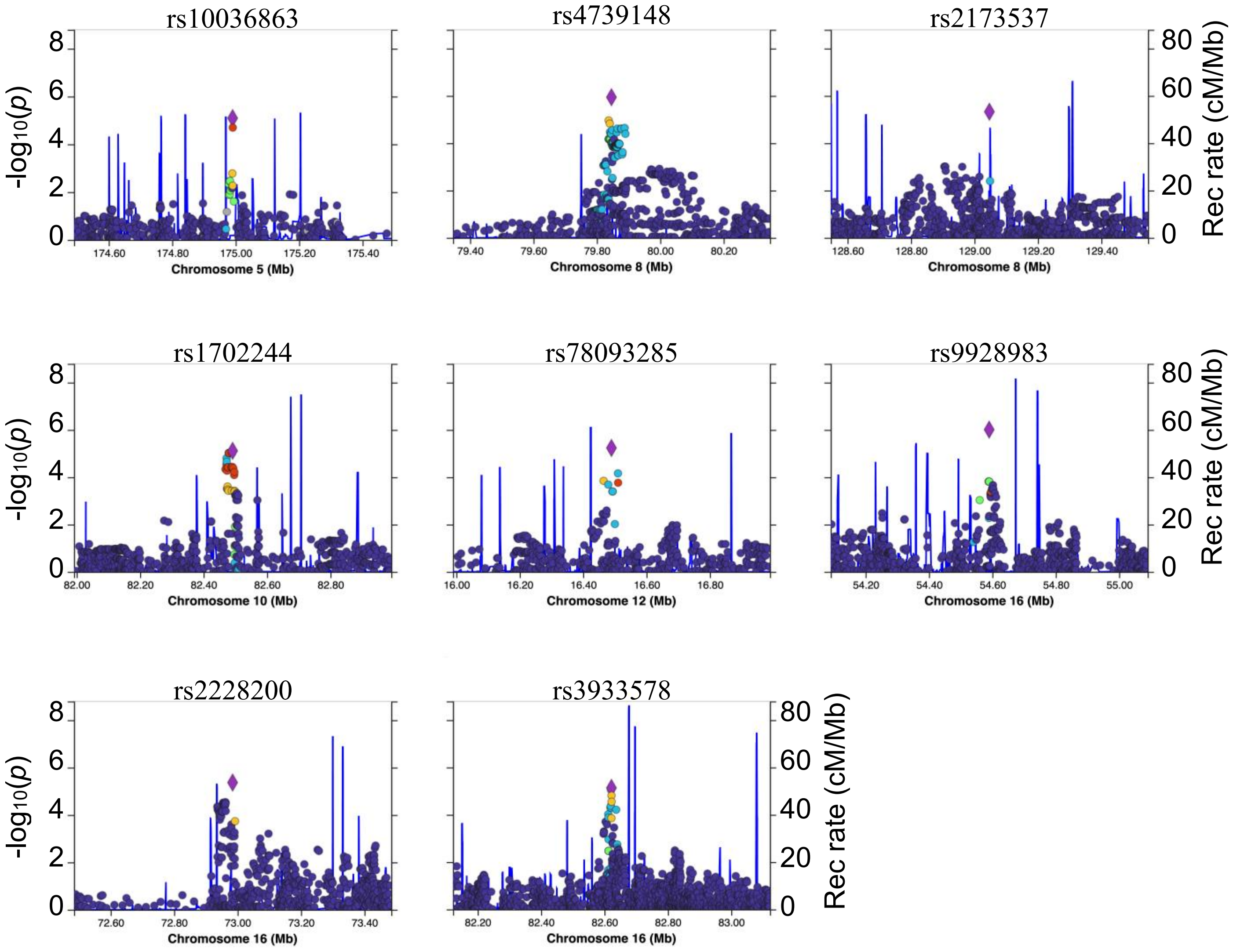

Supplemental Figure 10. Regional Manhattan plots at the vicinity of the selected SNPs.

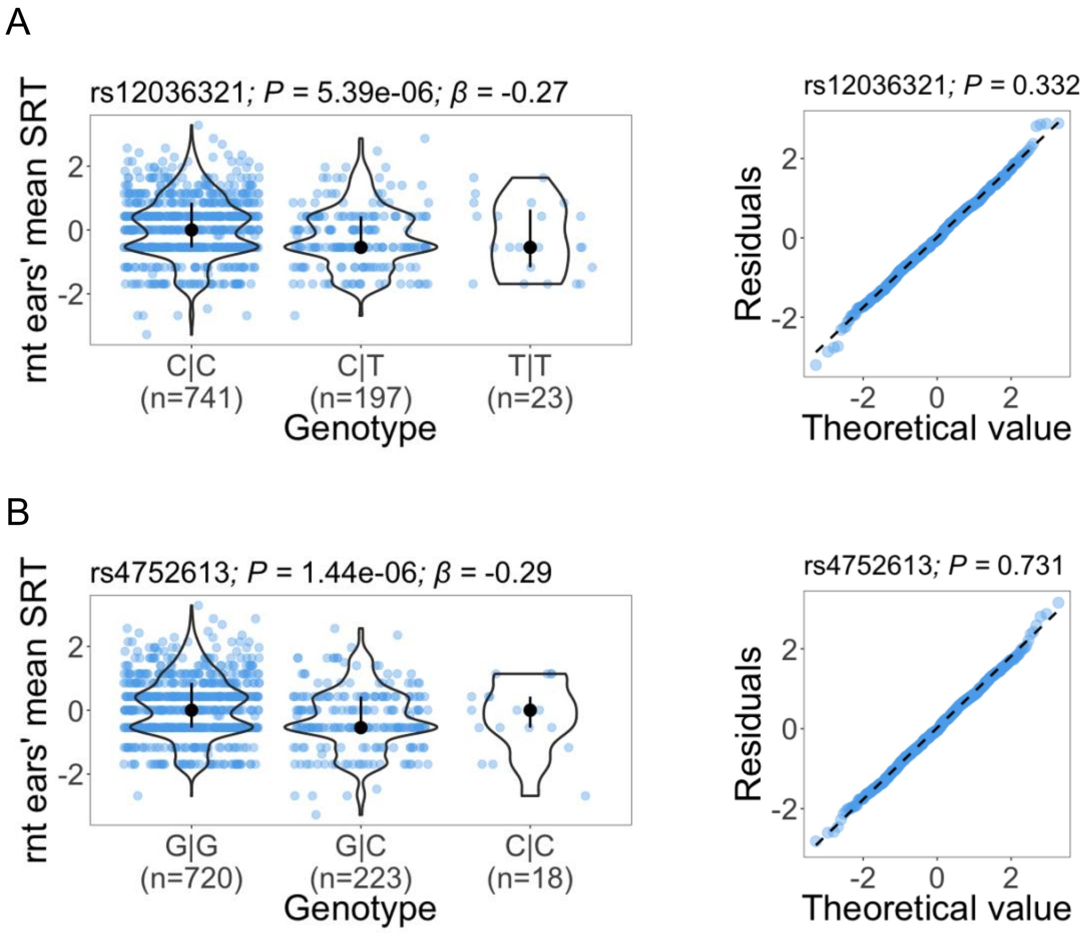

Supplemental Figure 11. Linear regression of rank normal transformed (rnt) SRT measurements relative to SNP genotypes. (A) rs12036321and (B) rs4752613. Left panels show the distribution of rank normal transformed SRT measurements relative to SNP genotypes. *P* values of linear regression models are provided. Right panels show the quantile-quantile plots of model's residuals with *P* values from the Shapiro–Wilk test for normality. Model: rntSRT = SNP genotype + age at audiometry + 10 population stratification PCs.
